# Genomic prediction in pigs using data from a commercial crossbred population: insights from the Duroc x (Landrace x Yorkshire) three-way crossbreeding system

**DOI:** 10.1186/s12711-023-00794-2

**Published:** 2023-03-28

**Authors:** Siyi Liu, Tianxiong Yao, Dong Chen, Shijun Xiao, Liqing Chen, Zhiyan Zhang

**Affiliations:** National Key Laboratory for Swine Genetics, Breeding and Production Technology, Jiangxi Agricultural University, Nanchang, 330045 China

## Abstract

**Background:**

Genomic selection is widely applied for genetic improvement in livestock crossbreeding systems to select excellent nucleus purebred (PB) animals and to improve the performance of commercial crossbred (CB) animals. Most current predictions are based solely on PB performance. Our objective was to explore the potential application of genomic selection of PB animals using genotypes of CB animals with extreme phenotypes in a three-way crossbreeding system as the reference population. Using real genotyped PB as ancestors, we simulated the production of 100,000 pigs for a Duroc x (Landrace x Yorkshire) DLY crossbreeding system. The predictive performance of breeding values of PB animals for CB performance using genotypes and phenotypes of (1) PB animals, (2) DLY animals with extreme phenotypes, and (3) random DLY animals for traits of different heritabilities (${h}^{2}$ = 0.1, 0.3, and 0.5) was compared across different reference population sizes (500 to 6500) and prediction models (genomic best linear unbiased prediction (GBLUP) and Bayesian sparse linear mixed model (BSLMM)).

**Results:**

Using a reference population consisting of CB animals with extreme phenotypes showed a definite predictive advantage for medium- and low-heritability traits and, in combination with the BSLMM model, significantly improved selection response for CB performance. For high-heritability traits, the predictive performance of a reference population of extreme CB phenotypes was comparable to that of PB phenotypes when the effect of the genetic correlation between PB and CB performance (${r}_{pc}$) on the accuracy obtained with a PB reference population was considered, and the former could exceed the latter if the reference size was large enough. For the selection of the first and terminal sires in a three-way crossbreeding system, prediction using extreme CB phenotypes outperformed the use of PB phenotypes, while the optimal design of the reference group for the first dam depended on the percentage of individuals from the corresponding breed that the PB reference data comprised and on the heritability of the target trait.

**Conclusions:**

A commercial crossbred population is promising for the design of the reference population for genomic prediction, and selective genotyping of CB animals with extreme phenotypes has the potential for maximizing genetic improvement for CB performance in the pig industry.

**Supplementary Information:**

The online version contains supplementary material available at 10.1186/s12711-023-00794-2.

## Background

With the advent of the genomic era, genomic prediction has become a mainstream selection technique in plant and animal breeding since it was first proposed [1–4]. Currently, the commercial livestock breeding industry relies on genome-wide dense markers to select desirable purebreds (PB). The candidate population with only genotypic records is predicted using data from a reference population with genotypic and phenotypic records, and the genetic merit of candidates is judged by their estimated breeding values (EBV) to perform selection and retention [1]. The typical process for genomic prediction in animal breeding is to select a certain number of PB as a reference population for phenotypic measurement, genotype the reference and candidate populations, and then to construct a statistical model to calculate the EBV of the candidate population [5, 6]. Currently, the GS processes are mainly implemented at the PB nucleus population level.

The ultimate goal of breeding is to improve the performance of commercial crossbreds (CB). Genetic progress created by selection for PB performance is reflected in commercial CB progeny after expansion and crossbreeding. However, because of differences in genetic background [7, 8], genotype-by-environment interactions [9, 10] and measurement methods [11], PB and CB performances differ for most traits. Previous studies have shown that the genetic correlation between PB performance and CB performance (${r}_{pc}$) for different trait categories such as growth, meat amount, meat quality, feed, and fertility is 0.6 on average [12]. Consequently, genomic selection based on PB performance amounts to indirect selection and the CB progeny of the best PB animals based on PB performance may not have the best performance [13]. In order to improve the accuracy of prediction of PB for CB performance, combined CB and PB selection (CCPS) was proposed [14]. Substantial gains can be achieved with CCPS compared to using only PB performance when ${r}_{pc}$ is lower than 0.7 [15]. In addition, Dekkers et al. [16] demonstrated that marker-assisted selection or genomic selection with marker effects estimated from CB performance can contribute to a significant increase in genetic gain. It has been demonstrated that the inclusion of CB data in the genetic evaluation of PB selection candidates contributes to improving the performance of hybrid progeny [17–23] because it can account for the genetic differences between PB and CB and the potential effect of genotype-by-environment interactions.

However, application of GS using CB performance has been limited and breeding companies are not inclined to use CB performance exclusively for genomic prediction. On the one hand, the acquisition of substantial CB data is demanding and time-consuming, as CB animals are usually not individually identified, and the systematic recording of CB phenotypes is challenging. On the other hand, although many studies have provided evidence for the effectiveness of using CB performance for prediction, most are based on fully simulated data [17–21] and suggest that some of the PB information should be retained in the reference population, requiring continued phenotyping in the PB nucleus population [15, 21, 24]. Therefore, detailed guidance on adopting genomic prediction using only CB information in a crossbreeding system is lacking.

Recently, with the rapid development of high-throughput phenotyping platforms, such as imaging spectroscopy and structured light sensors, it has become possible to automatically perform integrated measurements of livestock on a large-scale and within a short time [25, 26]. Thus, measuring CB performance of commercial herds that will be slaughtered and marketed uniformly, is technically feasible. Compared with the nucleus, massive phenotypes on commercial animals can be obtained more conveniently and more quickly in pre-market slaughter processing. Current research on the use of two-way CB performance for genomic prediction in pigs is comprehensive [22, 23], but studies on the predictive effectiveness of CB performance in a three-way crossbreeding system on breeding values of the parental PB from which they originated are lacking. Three-way crosses are the mainstream of crossbreeding in pigs, and the most common commercial pig in China is the Duroc x (Landrace x Yorkshire) pig (DLY). In this study, we designed a three-way crossbreeding system of commercial DLY pigs based on real PB ancestors, with subsequent hybridizations consistent with the actual production pattern. Because it is not feasible to genotype the entire commercial population, the fraction that is selected for genotyping should represent as much of the ancestral lineage as possible. Several studies have revealed that, compared to random selection of CB to genotype [27, 28], selective genotyping can improve prediction accuracy, and also that genotyping the individuals that deviate from the population mean can increase the efficiency of genome-wide association studies (GWAS) for quantitative traits [29, 30]. Thus, the idea of performing genomic selection based on CB performance can be optimized to use only CB with extreme phenotypes for prediction. Thus, the focus of our study was mainly on the use of CB with extreme phenotypes. Our objective was to compare the predictive performance of PB and CB phenotypes for prediction of breeding values of PB for CB performance for traits with different heritabilities and parental breeds of origin across different genetic evaluation models and population sizes. Furthermore, we explored the effect of genetic relationships between the reference and candidate populations on prediction accuracy and the potential of the different scenarios to detect and identify quantitative trait loci (QTL). The results provide comprehensive guidelines for the implementation of genomic prediction based exclusively on CB performance in practical crossbreeding systems from the perspective of the DLY three-way crossbreeding system.

## Methods

### Ancestral population

We calculated identity-by-descent (IBD) distances between individuals within a base population including 4074 Duroc pigs, 845 Landrace pigs, and 3616 Yorkshire pigs and selected 690 unrelated individuals including 60 Duroc pigs (10 boars and 50 sows), 110 Landrace pigs (10 boars and 100 sows), and 520 Yorkshire pigs (20 boars and 500 sows) to form the ancestral great-grandparent (GGP) generation GGP1. Within each breed, the pigs were unrelated to each other to ensure lineage diversity in the ancestral population, and all pigs met the breeding standard of good health, growth and appearance. The pigs were part of a collaborative genomic selection project built by Henan Xinda Livestock Co., Ltd. (Henan Province, China) and Jiangxi Agricultural University (Jiangxi Province, China). The base population from which these pigs originate are PB herds raised by the company for breeding purposes. These GGP1 pigs were genotyped with the CC1 Porcine single nucleotide polymorphism (SNP)50 BeadChip [31]; the genotypes were filtered to ensure a call rate higher than 0.95 and a minor allele frequency (MAF) higher than 0.01, and only SNPs that mapped to the *Sus scrofa* build 11.1 were included. In total, 39,755 SNPs passed the filters and were used in the simulation of subsequent generations, as described below.

### Simulated breeding system

**Fig. 1:**
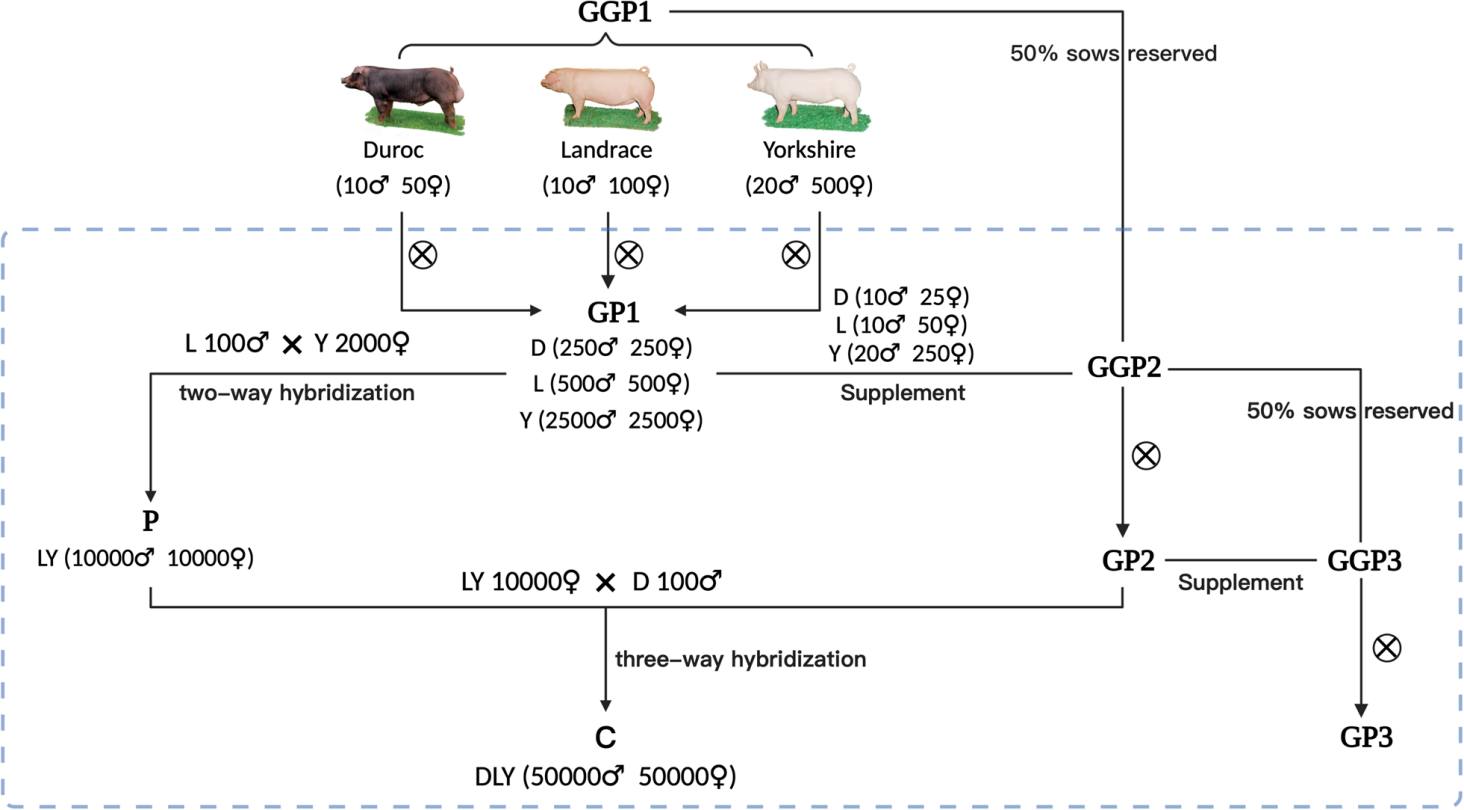
General overview of the DLY crossbreeding system. The GGP1 population was based on actual data, and the populations in the blue dashed box were the subsequent generations that were simulated. The symbol "⊗" represents within-breed mating

The subsequent generations in the simulated breeding system were generated as follows (see Fig. 1). The first grandparent (GP) generation was produced by within-breed mating of one male to many females GGP1 for expansion (10 males to 50 females for Duroc, 10 males to 100 females for Landrace and 20 males to 500 females for Yorkshire), producing 10 progeny per female (5 males and 5 females). After this step, 6500 GP1 individuals (500 Duroc, 1000 Landrace and 5000 Yorkshire with a male–female rate of 1:1) were obtained. Then, 100 Landrace boars and 2000 Yorkshire sows were randomly selected from the GP population and mated, producing 20,000 two-way hybrid Landrace x Yorkshire (LY) pigs (10,000 males and 10,000 females) in the parent (P) generation. The resulting 10,000 LY females were mated to 100 Duroc boars that were randomly selected from the GP population to produce 100,000 three-way hybrid DLY pigs (50,000 males and 50,000 females) in the commercial (C) generation.

The nucleus group was updated twice as hybridizations proceeded in the whole breeding system. The first nucleus PB group consisted of the GGP1 and the GP1. The role of the GP population is to update and supplement the GGP population and to provide PB individuals for subsequent hybridization. The replacement rate of the GGP was 100% for males and 50% for females and the number of individuals remained unchanged during the regenerations. We selected 35 Duroc pigs (10 boars and 25 sows), 60 Landrace pigs (10 boars and 50 sows), and 270 Yorkshire pigs (20 boars and 250 sows) from the GP1 population to update the GGP1. These GP1 individuals were drawn from the progeny of each GGP1 family to ensure that the selected GP individuals covered all the lineages of the original GGP evenly. The 50% of females that remained in GGP1 and individuals supplemented by GP1 formed the GGP2. The GP2 population was obtained by within-breed mating of GGP2 while the parent generation was produced, and the nucleus group was updated to the second generation. Then, 100 Duroc boars were randomly selected from GP2 as terminal sires. Parallel to the production of DLY commercial pigs, the GP3 population was generated. This breeding system did not involve directional selection, and no assessment of genetic merit for commercial CB performance was undertaken in the nucleus groups.

### Simulated genotypes and phenotypes

Genotypes of offspring were simulated based on the genotypes of the GGP1. Genotypes for the eligible SNPs (39,755) were phased in the GGP1 population using SHAPEIT v2.r837 [32]. The progeny genotypes were randomly sampled from the male and female gametic pools with 4 to 6 random crossovers on each chromosome and recombined into a new diploid, in which no interference was considered. Since the GGP1 individuals, which represented the evolution of each breed and the diversity among breeds, were genotyped, and given the short generation interval between GGP1 and the commercial generation, mutations were not considered in the progeny genotype simulation.

Phenotypes for quantitative traits controlled by 2000 QTL were simulated [1], which were randomly drawn from segregating loci in the GGP1 population. The effect of a QTL was sampled from a gamma distribution with a shape parameter of 0.4 [33]. Since the gamma distribution provides only positive effects, the sign of the QTL effect was sampled to be positive or negative with equal probability. The QTL effects were rescaled so that the genetic variance (${V}_{g}$) was equal to 1. We investigated the impact of genome prediction on traits with different heritabilities (${h}^{2}$), i.e. low (0.1), medium (0.3), and high (0.5). Environmental variance (${V}_{e}$) was determined based on the genetic variance and heritability (${h}^{2}=\frac{{V}_{g}}{{V}_{g}+{V}_{e}}$). The simulated QTL effects were multiplied by the allele counts of the causal loci (0, 1, or 2) and then summed over to calculate the true breeding value (TBV) of each animal. Finally, a standard normal residual effect was added to the TBV to obtain the phenotype of an individual for a quantitative trait with a specific heritability. The simulation processes were performed using the R software v3.4.3 and the genotype simulation was implemented using the R package SIMER (https://github.com/xiaolei-lab/SIMER).

### Reference and candidate populations

Selection is generally undertaken in the GP population, in which the breeding values of PB are estimated and ranked. The highest-ranked GP individuals are then selected to renew the GGP population for the next crossbreeding. The GP3 population is the target population, which should determine the genetic merit of the next nucleus group but, in practice, genotyping the 6500 GP3 individuals is not affordable. Thus, in this study, we selected four pigs (2 males and 2 females) from the progeny of each GGP3 sow as the candidate population, i.e. in total 2600 purebreds (200 Duroc, 400 Landrace, and 2000 Yorkshire).

The aim of this study was to explore the impact of using commercial CB performance for the selection of PB candidates. We focused on the impact of using records of CB that were selected based on extreme phenotypes versus CB that were randomly selected for prediction. Conventional genomic selection is performed within the nucleus group, usually using the PB performance of the previous generation to predict the genetic potential of the next generation, i.e., in this case, GGP3 would be used to predict GP3. Here, the limited number of individuals in GGP3 did not allow the influence of the reference population size on prediction to be evaluated. Therefore, the GP2 population, i.e. the previous nucleus group, was used to represent the conventional scheme. Varying numbers (500, 1000, 2000, 3000, 4000, 5000, 6000, 6500) of individuals used as the reference population were selected based on phenotype using one of the following three scenarios: (a) two-tailed extreme DLY crossbreds; (b) randomly selected DLY crossbreds; and (c) randomly selected GP2 purebreds. Fifty replicates were carried out for scenarios involving random selection.

### Genomic prediction of breeding values

The following single-trait mixed linear model was fitted to estimate the variance components and the breeding values: $\mathbf{y}=\mu +\mathbf{g}+\mathbf{e}$, where $\mathbf{y}$ is a vector of phenotypic records of the reference population for a particular trait, $\mu$ is the overall mean, $\mathbf{g}$ is a vector of random genetic effects, and $\mathbf{e}$ is a vector of random residual effects [34]. Two prediction models, genomic best linear unbiased prediction (GBLUP) and the Bayesian sparse linear mixed model (BSLMM), were used for genetic evaluation. In the GBLUP model, the genetic values correspond to the random effect, which is represented by $\mathbf{y}=\mu +\mathbf{Z}\mathbf{u}+\mathbf{e}$, where $\mathbf{u}$ is a vector of breeding values of all individuals, following the distribution ${\mathbf{u}\sim N(\boldsymbol{0},\mathbf{G}\sigma }_{u}^{2}$), $\mathbf{Z}$ is an incidence matrix relating observations in $\mathbf{y}$ to the corresponding random genetic effects, ${\sigma }_{u}^{2}$ is the additive genetic variance, $\mathbf{G}$ is the genomic relationship matrix based on SNP genotypes of the animals, which was calculated using VanRaden's method [35] as: $\mathbf{G}=\frac{{\mathrm{MM}}^{\mathrm{^{\prime}}}}{2\sum_{\mathrm{i}=1}^{\mathrm{n}}{p}_{i}{q}_{i}}$; the GBLUP analysis was implemented using the HiBLUP software [36].

The BSLMM model implemented in GEMMA [37] was $\mathbf{y}=\mu +\mathbf{X}{\varvec{\upbeta}}+\mathbf{e}$, where $\mathbf{X}$ is the centered genotype matrix, ${\varvec{\upbeta}}$ is the vector of allele substitution effects of the analyzed SNPs. The SNP effects were assumed to distributed as follows [38]:

$$\beta_{ i} |{\sigma }_{\beta_{ i}}^{2}=\left\{\begin{array}{c}\sim N\left(0,{\sigma }_{a}^{2}+{\sigma }_{b}^{2}\right) \text{with probability }\pi \\ \sim N\left(0,{\sigma }_{b}^{2}\right) \text{with probability} \,\left(1-\uppi \right)\end{array}\right.,$$

where ${\sigma }_{a}^{2}$ is the variance for the SNPs with large effects, ${\sigma }_{b}^{2}$ is the variance for the SNPs with minor effects, and $\uppi$ denotes the proportion of SNPs having large effects. Monte-Carlo Markov chains (MCMC) of length 200,000 with 10% discarded as burn-in were conducted to generate the posterior distributions. To obtain the EBV of the candidate population, the SNP genotypes (coded as 0, 1, 2) of individuals were multiplied by the estimated marker effects for each scenario: $\mathrm{GEBV}=\sum_{\mathrm{i}=1}^{\mathrm{n}}({\beta }_{i}\times {[\mathrm{SNPgenotype}]}_{\mathrm{i}}$). The prediction accuracy of the different scenarios and models was evaluated as the Pearson correlation coefficient between GEBV and TBV of the candidate population [39]. We also calculated the average TBV of the top 10% candidates (Best10%_TBV) based on GEBV to reflect the response to selection. In addition to the overall accuracy, the prediction accuracy within each breed was also extracted from the results for each training scheme. Scenarios were averaged over 50 replicates.

### Investigation of identity-by-state and identity-by-descent

Genetic relatedness and similarity between populations were evaluated by identity-by-state (IBS) and identity-by-descent (IBD). The IBS and IBD measures were calculated based on SNPs for individual pairs between the reference population and candidate population, as well as between the reference population and the GGP1 ancestral population. Pairwise comparisons of IBS and IBD were implemented using the software PLINK v1.90 [40] with the parameter "*--distance ibs square allele-ct*" for IBS and the parameter "*--genome*" for IBD. The SNPs were first pruned with a sliding window of 50 SNPs, a window step size of 10 SNPs, and a maximum ${\mathrm{r}}^{2}$ threshold of 0.2 to strive for independence between SNPs in the calculation. Scenarios with reference population sizes ranging from 500 to 6500 individuals with a trait with a heritability of 0.5 were used for comparison. The average IBS/IBD of all individuals in the reference population to each individual in the target population (GGP1/Candidate) was recorded.

### Genome-wide association studies (GWAS)

To investigate the potential of scenarios with different reference populations to detect and identify QTL, GWAS analyses were performed using a linear mixed model implemented in GEMMA v0.98 [37]. The significance threshold for the GWAS was determined with Bonferroni correction for multiple testing. For further validation, genome-wide pairwise LD between SNPs and QTL was evaluated for each scenario using the PopLDdecay software v3.41 [41] and pairwise LD with an ${\mathrm{r}}^{2}$ greater than 0.6 indicated a high degree of linkage disequilibrium between SNPs and QTL. In addition, we identified the peak SNP that reached the GWAS significance threshold within the 500 kb region upstream and downstream of each QTL and calculated the phenotypic variance explained (PVE) by the peak SNP, as follows [42]:

$${\text{PVE}}_{\text{i}}= \frac{2{a}_{i}^{2}{p}_{i}(1-{p}_{i})}{2{a}_{i}^{2}{p}_{i}(1-{p}_{i})+2\mathrm{N}{Se}_{({a}_{i})}^{2}{p}_{i}(1-{p}_{i})},$$

where the left-hand side of the equation is the PVE of the $i$th significant SNP, ${a}_{i}$ is the estimated size of the effect for SNP $i$ in the GWAS output, ${Se}_{({a}_{i})}$ is the standard error of effect estimate, ${p}_{i}$ is the minor allele frequency, and $\mathrm{N}$ is the sample size of the reference population. The PVE of the most significant SNP within each QTL region were summed to obtain the corresponding total significant PVE of the reference population (sigPVE).

## Results

### Overview of simulated traits

After scaling the QTL effects across the whole system (140,190 pigs), the overall genetic variance was set to 1. Statistics for the resulting TBV and phenotypes for the populations used for training are in Table 1. Traits with different heritabilities were created based on three levels of environmental variance while keeping the TBV unchanged. Compared to GP2, the average TBV of the commercial DLY population increased by 17.42%, which confirmed the utility of crossbreeding [43]. The DLY_extreme populations with different sample sizes were determined by sorting individuals based on phenotype and then taking half of the individuals from the top and bottom of the distribution, respectively. The average TBV under three heritabilities was similar for all extreme sampling schemes and did not differ significantly from the average TBV of the entire DLY population.

**Table 1:** Summary of true breeding values (TBV) and of phenotypic values in GP2, DLY, and DLY_extreme with different sampling sizes for traits of different heritabilities (${{h}}^{2}$)

Population	Size	TBV			Phenotype		
		${{h}}^{2}$= 0.5	${{h}}^{2}$= 0.3	${{h}}^{2}$ = 0.1	${{h}}^{2}$ = 0.5	${{h}}^{2}$ = 0.3	${{h}}^{2}$ = 0.1
GP2	6500	1.579 ± 1.040	1.579 ± 1.040	1.579 ± 1.040	1.580 ± 1.443	1.541 ± 2.505	1.405 ± 8.991
DLY	100,000	1.854 ± 0.968	1.854 ± 0.968	1.854 ± 0.968	1.854 ± 1.395	1.861 ± 2.532	1.823 ± 9.011
DLY_extreme	6500	1.878 ± 1.674	1.860 ± 1.233	1.872 ± 0.990	1.873 ± 3.163	1.853 ± 5.734	1.815 ± 20.385
	6000	1.879 ± 1.694	1.861 ± 1.245	1.871 ± 0.992	1.873 ± 3.206	1.853 ± 5.811	1.812 ± 20.659
	5000	1.877 ± 1.736	1.867 ± 1.263	1.870 ± 0.998	1.875 ± 3.302	1.854 ± 5.984	1.803 ± 21.271
	4000	1.879 ± 1.791	1.865 ± 1.277	1.875 ± 0.996	1.877 ± 3.415	1.855 ± 6.192	1.786 ± 21.997
	3000	1.883 ± 1.854	1.859 ± 1.298	1.880 ± 1.017	1.881 ± 3.557	1.853 ± 6.451	1.757 ± 22.908
	2000	1.882 ± 1.944	1.855 ± 1.345	1.890 ± 1.002	1.885 ± 3.751	1.845 ± 6.804	1.724 ± 24.135
	1000	1.861 ± 2.088	1.875 ± 1.422	1.884 ± 0.985	1.886 ± 4.067	1.839 ± 7.372	1.630 ± 26.158
	500	1.874 ± 2.236	1.892 ± 1.471	1.912 ± 0.989	1.883 ± 4.371	1.823 ± 7.900	1.454 ± 28.090

The form of TBV and phenotype was mean ± sd in the corresponding population

When the heritability of the trait decreased from 0.5 to 0.3, the average phenotype was not affected for any population, but a decline was observed when heritability decreased from 0.3 to 0.1, especially in the GP2 and DLY_extreme populations of small sizes. The standard deviations (SD) of phenotypes in the GP2 and DLY populations were approximately equal to the corresponding environmental variance, indicating that the simulated traits were realistic. Compared with the whole DLY population, the SD of the phenotypes was much larger for the DLY_extreme population, and increased as heritability and population size decreased. The investigated sampling strategies can explore the contribution of CB that deviate from the population mean for genomic prediction [44].

### Overall accuracy of genomic prediction

**Fig. 2:**
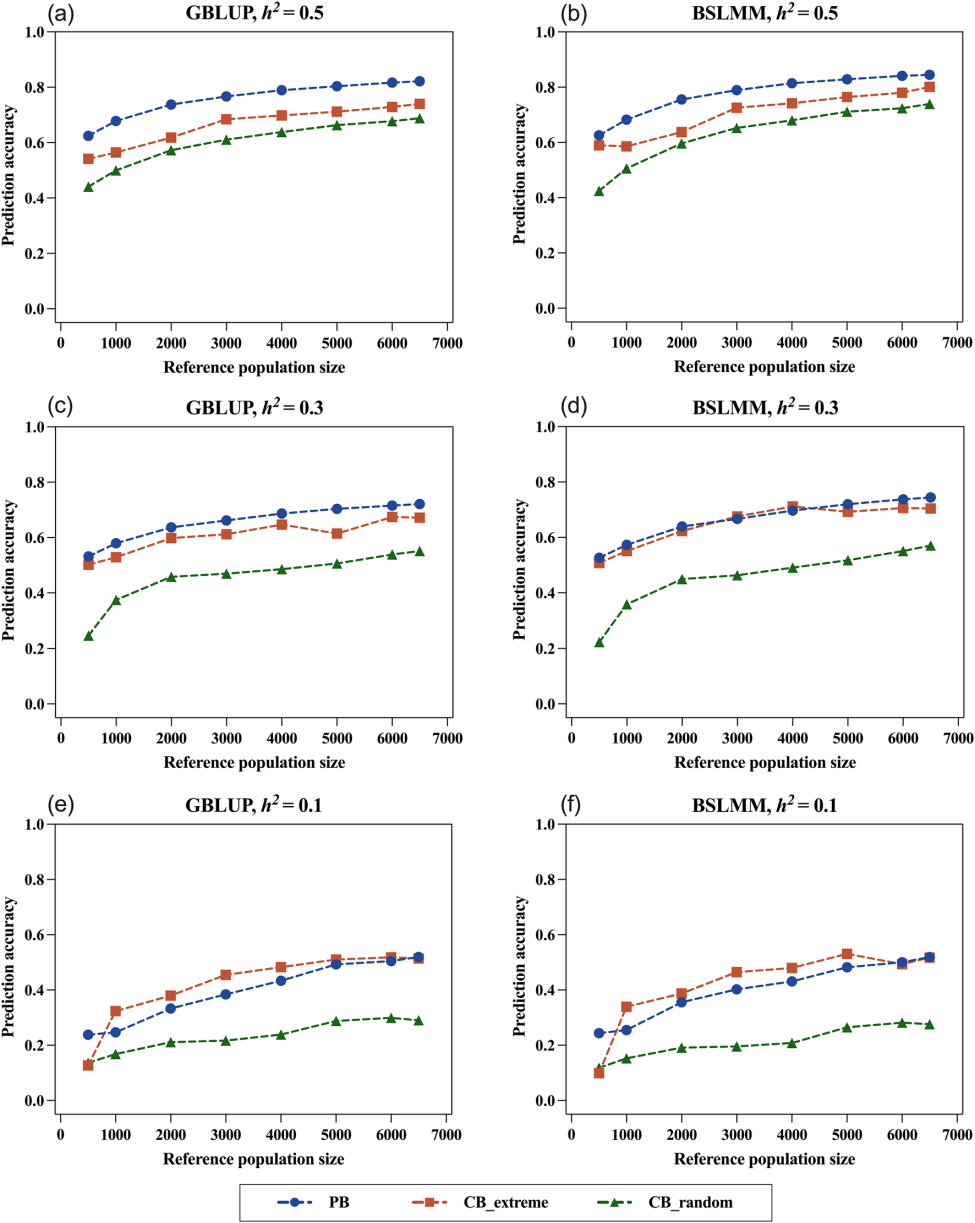
Predicted accuracy of estimated breeding values of the whole candidate population for different reference population types (PB: purebreds in GP2; CB_extreme: two_tailed crossbreds in DLY; CB_random: random crossbreds in DLY) and sizes (500, 1000, 2000, 3000, 4000, 5000, 6000, 6500). **a** GBLUP method, ${h}^{2}$ = 0.5, **b** BSLMM method, ${h}^{2}$ = 0.5, **c** GBLUP method, ${h}^{2}$ = 0.3, **d** BSLMM method, ${h}^{2}$ = 0.3, **e** GBLUP method, ${h}^{2}$ = 0.1, **f** BSLMM method, ${h}^{2}$ = 0.1. Predicted accuracy was averaged across 50 replications for scenarios involving randomization

The accuracy of GEBV for the different training programs and models for traits with different heritabilities was measured in the whole candidate population and the results are shown in Fig. 2. The average TBV of the best 10% candidates based on GEBV (Best10%_TBV) was calculated and is provided in Table 2. All genetic variance components were estimated for each scenario. As expected, the overall prediction accuracy increased as the size of the reference population increased, and the predictability of traits increased as the heritability increased. Compared to GBLUP, using the BSLMM model for training slightly improved prediction accuracy (Fig. 2 and Table 2). The two CB populations from the same commercial group but corresponding to different sampling strategies showed significant differences in the prediction of PB in the candidate population, i.e. for the random sampling strategy (CB_random), the overall prediction accuracy was lowest, but for the extreme phenotypic subset (CB_extreme) it was comparable to that of the GP2 population (PB).

**Table 2:** Average true breeding value (TBV) of the top 10% candidates based on genomic breeding values predicted by different reference populations (PB: purebreds in GP2; CB_extreme: two_tailed crossbreds in DLY; CB_random: random crossbreds in DLY) with different population sizes (500, 1000, 2000, 3000, 4000, 5000, 6000, 6500) and two methods (GBLUP and BSLMM) for different heritabilities (${{h}}^{2}$)

${{h}}^{2}$	Class	GBLUP			BSLMM		
		PB	CB_extreme	CB_random	PB	CB_extreme	CB_random
0.5	6500	2.943	2.771	2.730	3.016	3.026	2.874
	6000	2.932	2.746	2.708	3.019	2.951	2.844
	5000	2.899	2.715	2.689	2.984	2.817	2.820
	4000	2.863	2.683	2.648	2.950	2.817	2.758
	3000	2.810	2.640	2.603	2.882	2.766	2.720
	2000	2.734	2.655	2.522	2.793	2.747	2.599
	1000	2.593	2.488	2.410	2.614	2.587	2.438
	500	2.465	2.481	2.333	2.468	2.601	2.313
0.3	6500	2.678	2.640	2.481	2.720	2.821	2.544
	6000	2.653	2.592	2.480	2.697	2.848	2.513
	5000	2.617	2.477	2.419	2.668	2.765	2.457
	4000	2.581	2.619	2.391	2.606	2.874	2.406
	3000	2.531	2.574	2.357	2.538	2.705	2.350
	2000	2.490	2.452	2.338	2.492	2.625	2.319
	1000	2.383	2.365	2.241	2.374	2.407	2.218
	500	2.302	2.326	2.102	2.296	2.331	2.040
0.1	6500	2.202	2.589	2.126	2.246	2.630	2.092
	6000	2.200	2.576	2.152	2.216	2.585	2.111
	5000	2.231	2.543	2.153	2.206	2.597	2.109
	4000	2.162	2.524	2.090	2.165	2.451	2.033
	3000	2.209	2.454	2.060	2.173	2.469	2.000
	2000	2.160	2.160	2.002	2.142	2.154	1.962
	1000	2.137	2.170	1.920	2.051	2.191	1.864
	500	2.079	1.980	1.839	2.035	1.943	1.791

TBV were averaged across 50 replications for scenarios involving randomization

For high-heritability traits (${h}^{2}$ = 0.5), the prediction accuracy followed the same trend for both the GBLUP and BSLMM models, i.e. PB > CB_extreme > CB_random, and the difference in prediction accuracy between CB_extreme and PB was 0.094 and 0.069 under the GBLUP and BSLMM model, respectively (Fig. 2a and b). Genomic selection focuses not only on prediction accuracy but also on the response to selection, and the Best10%_TBV can reflect the response to selection. For the BSLMM model, the Best10%_TBV was highest when using the CB_extreme population of size 6500 (3.026) (Table 2). Corresponding to this scenario, the difference in prediction accuracy between the CB_extreme (0.801) and PB (0.845) populations was minimal (Fig. 2b). This implies that the CB_extreme population has the potential to select PB candidates with high TBV.

When predicting traits with medium heritability (${h}^{2}$ = 0.3), the average difference in prediction accuracy between the CB_extreme and PB populations under the GBLUP model decreased to 0.049 (Fig. 2c). This difference was even smaller for the BSLMM, for which the trends in accuracy for the PB and CB_extreme almost overlapped, as shown in Fig. 2d. Prediction accuracy of the PB population barely changed with BSLMM compared to that with GBLUP. In contrast, the CB_extreme population benefited from the BSLMM training method with an overall improvement in accuracy compared to GBLUP. The results of Best10_TBV for the candidate population showed that the Best10_TBV predicted by using the CB_extreme population was higher than that predicted by the PB population across all population sizes with the BSLMM model, and the highest Best10_TBV (2.874) was obtained by using the CB_extreme population of size 4000 (Table 2). When the size of the reference population was 4000, the prediction accuracy of the CB_extreme population with BSLMM was 0.712, which was slightly higher than that obtained with the PB population (0.697). For the prediction of medium-heritability traits, the use of the CB_extreme population with the BSLMM training method can achieve higher accuracy than using the PB reference population.

The predictability of low-heritability traits was modest due to the excessive influence of non-genetic factors. Remarkably, the CB_extreme population showed better predictive potential than the PB population for both models, both in terms of prediction accuracy (Fig. 2e and f) and Best10_TBV (Table 2). Accurate prediction of breeding values of PB candidates requires a larger reference population for low-heritability traits than for medium-heritability traits but the CB_extreme population of size 5000 was sufficient to obtain a relatively high prediction accuracy. Among the three reference populations, CB_extreme was the most optimal predictor for low-heritability traits.

### Prediction accuracy within each pure breed

**Fig. 3:**
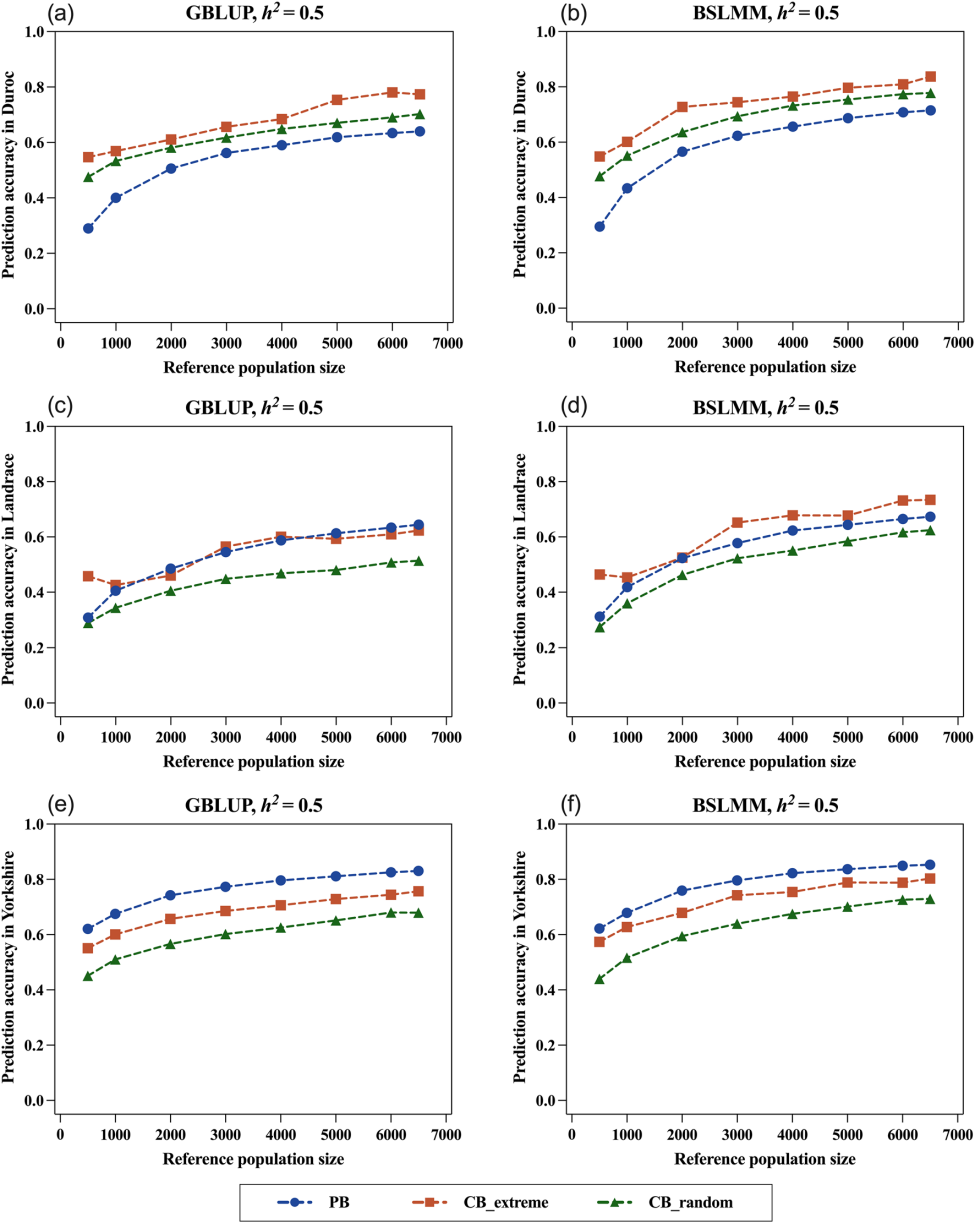
Predicted accuracy of estimated breeding values within the candidate population for each pure breed for different reference population types (PB: purebreds in GP2; CB_extreme: two_tailed crossbreds in DLY; CB_random: random crossbreds in DLY) and sizes (500, 1000, 2000, 3000, 4000, 5000, 6000, 6500). The results are shown for a trait with a heritability of 0.5. The accuracies of prediction for Duroc (**a** and **b**), Landrace (**c** and **d**), and Yorkshire (**e** and **f**) within the candidate population are shown separately. The results of the GBLUP method (**a**, **c**, and **e**) and the BSLMM method (b, d, and f) are presented. Predicted accuracy was averaged across 50 replications for scenarios involving randomization

**Fig. 4:**
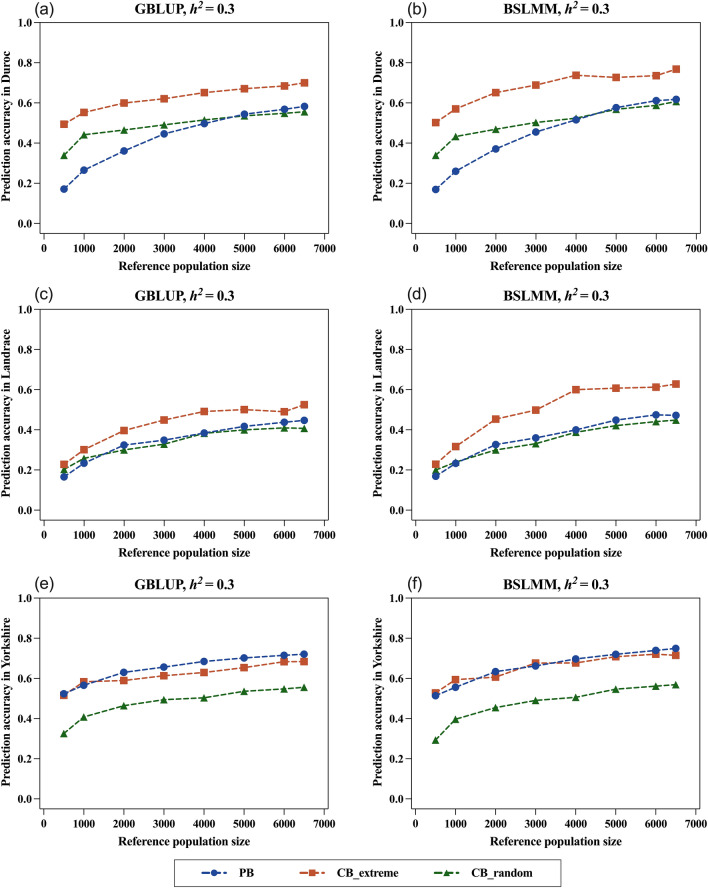
Predicted accuracy of estimated breeding values within the candidate population for each pure breed for different reference population types (PB: purebreds in GP2; CB_extreme: two_tailed crossbreds in DLY; CB_random: random crossbreds in DLY) and sizes (500, 1000, 2000, 3000, 4000, 5000, 6000, 6500). The results are shown for a trait with a heritability of 0.3. The accuracies of prediction for Duroc (**a**, **b**), Landrace (**c**, **d**), and Yorkshire (**e**, **f**) within the candidate population are shown separately. The results of the GBLUP method (**a**, **c**, **e**) and the BSLMM method (**b**, **d**, **f**) were presented. Predicted accuracy was averaged across 50 replications for scenarios involving randomization

The number of animals from the three parental purebreds needed to produce the three-way hybrid DLY was varied. Since the ratios of purebreds in the candidate population were 1:2:10 (Duroc:Landrace:Yorkshire), the overall accuracy did not exactly reflect the selection response within each breed. Therefore, we extracted the prediction accuracy within each pure breed from the results predicted by different reference populations to determine the optimal selection scheme for each breed. The candidate population included 200, 400, and 2000 Duroc, Landrace, and Yorkshire individuals, respectively. The correlations between the TBV and GEBV for the candidates of each breed are in Figs. 3 and 4, and in Additional file 1: Fig. S1. Similarly, Best10_TBV was calculated for the candidate group of each breed (see Additional file 5: Table S1 and Additional file 6: Table S2).

As expected, the CB outperformed the PB for the prediction of the Duroc candidates since the CB were derived from the terminal paternal Duroc. For both models, the trends in accuracy for the three reference populations were: CB_extreme > CB_random > PB. The highest prediction accuracy for Duroc was always obtained by using the CB_extreme population combined with the BSLMM model, i.e. 0.837, 0.768, and 0.638 for the low-, medium- and high-heritability traits, respectively (Figs. 3b and 4b) and (see Additional file 1: Fig. S1b). The difference in prediction accuracy between the CB_extreme population and the two other reference populations increased as heritability of the trait decreased.

For the prediction of Landrace candidates, the CB_extreme population also showed good predictive performance. The trends in the prediction accuracy of the CB_extreme and PB populations almost overlapped when predicting high-heritability traits with GBLUP (Fig. 3c), but CB_extreme reached the highest Best10_TBV (3.314) at a reference population size of 4000 (see Additional file 5: Table S1). When BSLMM was used for training, the improvement in prediction accuracy was greater for the CB_extreme than for the PB population (Fig. 3d), and the highest Best10_TBV (3.389) was obtained for the CB_extreme population, even at a size of 3000 (see Additional file 6: Table S2). For medium- and low-heritability traits, CB_extreme consistently ranked at the top across all population sizes and for both models, both in terms of prediction accuracy and Best10_TBV.

The PB reference population was from GP2, which had the same Duroc:Landrace:Yorkshire ratios of 1:2:10 as the candidate population (GP3), with the Yorkshire individuals representing the largest proportion. When the GBLUP model was used for prediction, prediction accuracy for a breed increased with the number of individuals of the breed in the reference population [45, 46]. This was confirmed by the results of the prediction of Yorkshire candidates in the PB reference population using GBLUP (Figs. 3e and 4e). Notably, when BSLMM was used for the prediction of medium-heritability traits, the accuracy obtained by the CB_extreme population improved and became similar to that obtained with the PB population (Fig. 4f) and (see Additional file 6: Table S2). For low-heritability traits, the Best10_TBV was always greater when using the CB_extreme population than with the PB population for both models (see Additional file 6: Table S2). Although the prediction accuracy of the CB_extreme population was not superior when using GBLUP (see Additional file 1: Fig. S1e), it was significantly improved when using the BSLMM approach and even surpassed that of the PB population (see Additional file 1: Fig. S1f).

### Genetic relatedness

**Fig. 5:**
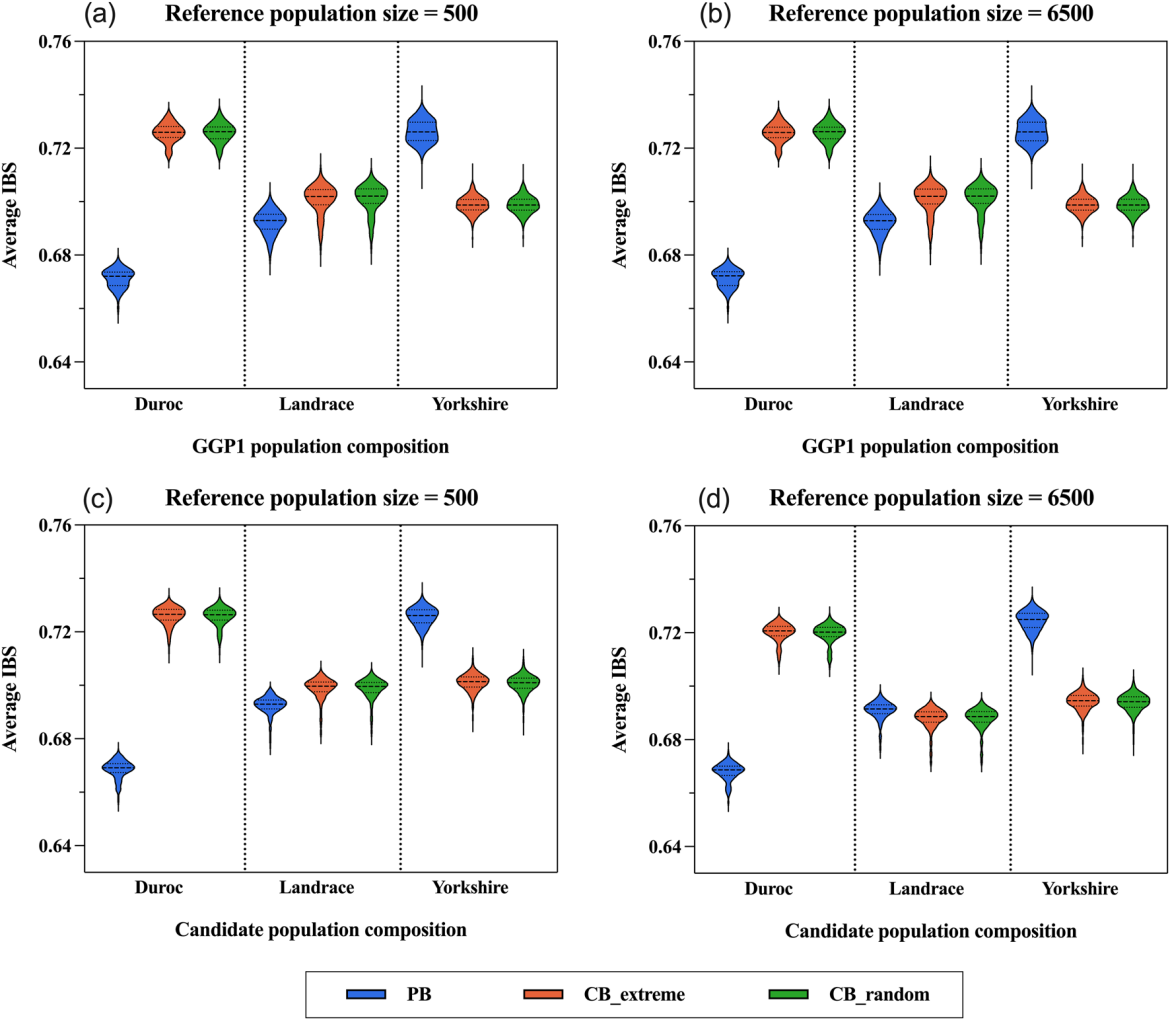
Pairwise IBS calculations between the reference population and the GGP1 ancestral population (**a**, **b**) and between the reference population and the candidate population (**c**, **d**). The violin distribution of mean IBS coverage of all individuals in the reference population against each individual in the target population is shown, where the x-axis denotes the breeds within the target population (GGP1/Candidate), and the y-axis is the mean IBS corresponding to each individual in the target population. The distributions of three reference population types (PB, CB_extreme, and CB_random) and two population sizes (500 for **a**, **c**, 6500 for **b**, **d**) are shown. The IBS coverage for each individual was averaged across 50 replications for scenarios involving randomization

The genetic relatedness and similarity of the different reference populations to the candidate population and to the GGP1 ancestral population were measured by IBS and IBD. The mean IBS/IBD of all individuals in the reference population to each individual in the target population (GGP1/Candidate) was calculated. The average IBS was plotted against the breed classification of the target population, as shown in Fig. 5. Since the calculation of IBS and IBD was based on SNP genotypes, and they are not affected by the phenotypes, the results for the CB_extreme and CB_random populations were identical, and both represented the performance of the commercial population, which in our study were combined as CB. Therefore, the different reference populations were grouped into two categories, PB and CB. Measurements of IBS and IBD can be used to compare the predictive potential of genetic relatedness-based methods, such as GBLUP [47]. No differences were observed in the average IBS of the GGP1 ancestral population between the smallest (Fig. 5a) and largest (Fig. 5b) reference population sizes for the three reference population designs. As expected, the IBS was higher for CB than for PB by an average of 0.055 for Duroc ancestors, and PB performed better when targeting GGP1_Yorkshire, on average by 0.027. When targeting GGP1_Landrace, the CB slightly exceeded the PB in average IBS. In general, CB were more closely related to Duroc and Landrace ancestors compared to PB, although the generation interval of PB to GGP1 was shorter than CB to GGP1.

Focusing on genetic relatedness with the candidate population, the performance of PB was not influenced by the size of the reference population. However, the average IBS of CB decreased slightly as the size of the reference population increased (Fig. 5c and d). Only the results for reference population sizes of 500 and 6500 are presented here, and the specific trend of average IBS for CB (from 500 to 6500) is shown in Additional file 2: Fig. S2. The average IBS between the reference population and candidate population can explain the prediction accuracy of the corresponding scenarios with GBLUP, although this is only valid when predicting high-heritability traits [48]. The high genetic similarity between DLY crossbreds in the reference population and Duroc purebreds in the candidate population validated the excellent performance of CB in the prediction of Duroc candidates, while the PB outperformed the CB in the prediction of Yorkshire candidates due to the greater genetic relatedness resulting from the larger number of Yorkshire individuals in the PB population. The average IBS for the Landrace candidates showed minimal difference between the PB and CB. Although the number of PB was stable and the number of CB declined slightly as the population size increased, overall they remained comparable. This corresponded to the results for the prediction of Landrace candidates in GBLUP (Fig. 3c), with slight fluctuations in prediction accuracy but no significant differences. Similarly, the results of average IBD also demonstrated the dominance of CB over Duroc and PB over Yorkshire in terms of genetic relationships (see Additional file 3: Fig. S3).

### QTL detection and linkage disequilibrium

**Fig. 6:**
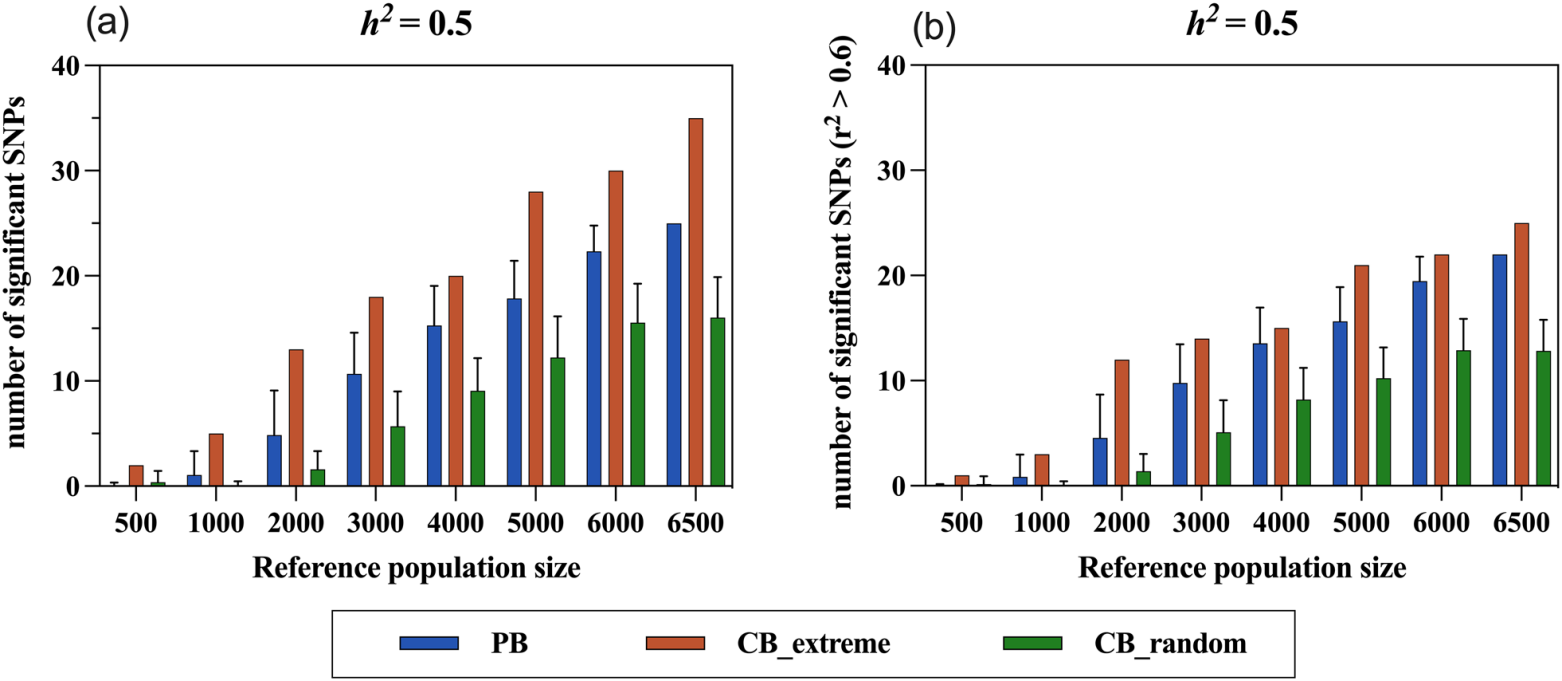
Number of SNPs significantly associated with the simulated traits in different reference population scenarios detected by GWAS (**a**) and number of significant SNPs with an ${\mathrm{r}}^{2}$ higher than 0.6 (**b**). The results are shown for traits with a heritability of 0.5, including the investigations of three reference population types (PB, CB_extreme, and CB_random) and eight reference population sizes (500, 1000, 2000, 3000, 4000, 5000, 6000, 6500). The scenarios involving randomization were averaged, and error bars denotes the SD between 50 replicates

To explore the efficiency of using PB versus CB to detect QTL, a GWAS was performed within each reference scenario based on 37,755 SNPs and simulated phenotypes. In addition, pairwise LD between 37,755 SNPs and 2000 QTL was estimated, and the combination with the highest LD was prioritized when a SNP was in LD with multiple QTL. First, we counted the number of SNPs that reached the genome-wide suggestive threshold for different reference populations and then we counted the number of significant SNPs that had an LD with the QTL higher than 0.6 (Fig. 6). Markers that reached the suggestive threshold and had an LD higher than 0.6 with the QTL were strong indicators for the identification of trait-associated QTL.

The results showed that the performance of the CB_extreme population in GWAS was superior to that of the PB and CB_random populations, with the largest number of detected significant SNPs (Fig. 6a) and of significant SNPs in high LD (Fig. 6b). For high-heritability traits, the number of detected SNPs increased with increasing population size but the overall trend remained: CB_extreme > PB > CB_random. Although both the random and the extreme phenotypic individuals came from the commercial CB population, they differed significantly in their potential to detect QTL, with roughly twice as many significant SNPs in high LD identified with the CB_extreme than with the CB_random population (Fig. 6b).

The results of the GWAS for the medium- and low-heritability traits showed that the potential to detect QTL of the PB and CB_random populations was almost 0, even at the largest population size. However, the advantage of the CB_extreme population was more evident for medium- and low-heritability traits, with 11 significant SNPs detected for the medium-heritability trait, 10 of which were highly linked to known QTL. For the low-heritability trait, three significant SNPs were detected by using the CB_extreme population, all in high LD (see Additional file 4: Fig. S4).

### Phenotypic variance explained by the peak SNPs

Based on the physical location of each QTL, upstream and downstream 500-kb intervals were classified as a QTL region (1 Mb long). We selected the peak SNP among those that reached the significance threshold in each QTL region and calculated the phenotypic variance explained by this SNP. The PVE of the peak SNPs were summed, as shown in Table 3, although significant signals were not detected in each QTL region. Each significant peak SNP can be used to quantify the impact of the QTL on the trait, although 100% representation is achieved only with complete LD between the peak SNP and QTL. The potential of the different populations to detect and identify QTL can be further validated based on the PVE of significant peak SNPs [49].

**Table 3:** Sum of the phenotypic variances explained (PVE) by the peak SNP within each QTL region in the GWAS results of different reference populations (PB: purebreds in GP2; CB_extreme: two_tailed crossbreds in DLY; CB_random: random crossbreds in DLY) with different population sizes (500, 1000, 2000, 3000, 4000, 5000, 6000, 6500) for traits of different heritabilities (${{h}}^{2}$) (%)

${{h}}^{2}$	Class	Reference population size							
		500	1000	2000	3000	4000	5000	6000	6500
0.5	PB	0.200	1.007	2.202	3.973	4.695	4.794	5.108	5.379
	CB_extreme	4.724	2.391	8.750	9.443	8.522	10.530	10.182	10.288
	CB_random	0.765	0.151	1.081	2.803	3.461	3.697	4.510	4.372
0.3	PB	0.409	0	0.025	0.069	0.072	0.058	0.032	0
	CB_extreme	0	0	2.727	2.297	3.392	2.624	2.494	2.888
	CB_random	0.092	0.049	0.028	0	0.038	0.165	0.201	0.323

PVE were averaged across 50 replications for scenarios involving randomization

As expected, the trend for the PVE in the GWAS results for high-heritability traits was the same as for the number of detected significant SNPs, i.e. CB_extreme > PB > CB_random (Table 3). However, we observed that the PVE of CB_extreme did not increase with population size, with the PVE of CB_extreme being higher with a population size of only 500 (4.7%) than with size of 1000 (2.4%). This may be attributed to the Beavis effect occurring in the CB_extreme population, also known as the “winner’s curse” [50]. When the evaluation was conducted in a small population, the small number of statistically significant QTL detected (Fig. 6) resulted in an inflated effect size for the identified QTL, thus an overestimation of the phenotypic variance of the significant peak SNPs associated with the identified QTL. Populations with larger sizes are more effective to detect QTL and, thus, the estimation of PVE is also more accurate. For CB_extreme, the PVE obtained from the evaluation with a population size of at least 2000 can reflect the actual magnitude of the associated QTL. The PVE of significant peak SNPs was about twice as large for the CB_extreme population (10.3%) than for the PB (5.4%) and CB_random (4.3%) populations at the largest population size of 6500, which might reflect the actual disparity between these three populations (Table 3). Since the potential of PB and CB_random to detect QTL for medium- and low-heritability traits (see Additional file 4: Fig. S4) was weak, the corresponding PVE was almost 0. In addition, the phenotypic variance captured by CB_extreme was also greatly reduced, with a maximum of only 3.4% for medium-heritability (Table 3) and 0.8% for low-heritability traits (see Additional file 7: Table S3).

## Discussion

The aim of this study was to investigate the effect of using commercial crossbreds as a reference population for genomic prediction in pigs, focusing on the performance of individuals with extreme phenotypes. The predictability of PB performance and CB performance for low-, medium-, and high-heritability traits was compared across different reference population sizes and prediction models. Furthermore, we explored the effect of genetic relatedness on prediction accuracy and the ability for GWAS to detect genetic variations associated with traits for the above scenarios.

Our results indicate that individuals with extreme phenotypes in the commercial population have a definite advantage for genomic prediction for medium- and low-heritability traits. In addition, predictions based on the BSLMM model resulted in greater responses to selection than the GBLUP model. For high-heritability traits, the performance of training on commercial crossbreds with extreme phenotypes was comparable to that of training on purebreds if the sampling size was large enough. Although similar prediction accuracies could be achieved by training on the same number of purebreds versus extreme phenotypic crossbreds, commercial crossbreds could provide more accessible phenotypic measurements and less threat to biosecurity. This study provides insights into genomic prediction for different target traits and different parental breeds in pig crossbreeding. Genotyping crossbreds with extreme phenotypes in commercial populations could facilitate an efficient and secure genomic selection.

### Breeding system for analysis

Combined crossbred and purebred selection has been an optimal solution to increase genetic response for genomic prediction, and several studies have investigated the proportion of CB to be included in the training population [15, 20, 21], as well as the use of different prediction models. [51, 52]. Here, we implemented the bottom-up selection strategy with a 100% CB inclusion rate in the reference population, refraining from phenotype collection in the nucleus group. Currently, with the development of livestock farming, many breeding companies have established the production pattern of nucleus-multiplication-commercial farms. Using high-throughput automated phenotyping techniques, it is convenient and fast to obtain multiple phenotypic records on commercial crossbreds. If the selection response based on PB information can be achieved by using CB information, the GS application based on CB performance is advantageous because of the easier phenotypic measurements and fewer biosafety concerns. However, it is impractical to collect genotypes and phenotypes for all crossbreds in the commercial group that are to be included in genetic evaluation. Instead, we focused on selective genotyping of individuals with extreme phenotypes, and CB performance of this subset was expected to substitute conventional PB performance for the selection of nucleus purebreds.

To date, many studies have evaluated the effect of CB performance on PB selection by using simulated data [17–20]. In our study, we used real genotyping data on pigs, which reflects the result of the formation of breeds and the authentic history of the evolution of the population and also embraced the real pattern of LD between loci in different breeds. Taking the most common commercial herds of the three-way hybrid DLY on the market as a breeding goal, we designed the construction of a 100,000 DLY pig population based on real PB ancestors (GGP1) with subsequent hybridizations conforming to the actual production patterns. The point of crossbreeding is the selection of PB, specifically the selection of individuals with excellent genetic merit from the GP to form the next GGP. The nucleus group was updated to the third generation (GGP3 + GP3) when commercial DLY pigs were generated. Given the timeliness of the breeding industry, our aim was to select outstanding individuals from GP3 to form the next generation of GGP (GGP4), with the expectation of generally improved performance in the commercial CB progeny produced by a new round of crosses based on GGP4.

### Prediction accuracy

Our results showed that, with GBLUP, the overall prediction accuracy of the CB_extreme population was lower than that of the PB reference population when predicting high- and medium-heritability traits, but that it can be enhanced by using the BSLMM model. However, the prediction accuracy of PB was overestimated in our study since ${r}_{pc}$ was assumed to be 1. The breeding values of PB candidates obtained by using CB performance for prediction is GEBV_CB_, while that by using PB performance is GEBV_PB_. Ultimately, we focused on the GEBV for CB performance of PB selection candidates, i.e., GEBV_CB_. A genetic correlation between PB and CB performance (${r}_{pc}$) can affect the prediction accuracy of PB reference populations [53]. Due to differences in genetic background, management and housing conditions, and trait measurements, ${r}_{pc}$ varies between traits [12]. The accurate evaluation of ${r}_{pc}$ requires a substantial amount of data for a given trait, and ${r}_{pc}$ values vary between different combinations of PB lines and CB progeny [54]. Thus, estimating a separate ${r}_{pc}$ for each unique PB-CB combination in a large and realistic breeding program is necessary. Traits in this experiment were based on three common levels of heritability without considering different combinations of heritability and ${r}_{pc}$, and the multiple possible levels of ${r}_{pc}$ for real traits. As a result, estimates of the prediction accuracy of PB here were biased upward for traits with an ${r}_{pc}$ lower than 1. When using the GBLUP model, the largest difference in prediction accuracy between CB_extreme and PB was 0.119 (reference size = 2000) and 0.089 (reference size = 5000) for high (0.5) and medium (0.3) heritability traits, respectively. Assuming a relatively high genetic correlation between PB performance and CB performance for simulated traits with an ${r}_{pc}$ of 0.8, the accuracy of PB would be reduced by 20% overall, corresponding to a reduction of 0.147 and 0.140 for the above scenarios. Therefore, since the ${r}_{pc}$ is lower than 1 and given the potential impact of ${r}_{pc}$ on the PB reference population, CB_extreme actually outperformed PB in the predictions of medium- and high-heritability traits, based either on GBLUP or BSLMM. Although simulated traits were simple and based on additive effects only, without considering non-additive effects and genotype-by-environment (G × E) interaction, which may lead to a slightly higher accuracy overall, the trends in prediction accuracy were not affected.

In this study, the predicted results of different sampling strategies in the commercial CB population were similar to those of previous studies [28, 55, 56], with selective genotyping of top and bottom crossbreds (CB_extreme) being distinctly better than random genotyping (CB_random). More genetic variations associated with traits are covered by genotypic information of individuals with extreme phenotypes compared to that of random individuals [29], which we also verified by GWAS. When prediction was done with the GBLUP model, we used the variance components that were estimated for each reference population scenario, as shown in Additional file 8: Table S4. Using true variance components contributes to unbiased predictions of GEBV in selective genotyping [56]. However, the true variance structure of traits is not accessible in practice. We found that variance components were greatly overestimated with extreme selective genotyping. In contrast, random genotyping, which represented the distribution of the whole commercial population, resulted in a more accurate estimate of the variance components, in agreement with previous studies [28, 57]. Maximizing the role of the commercial CB population by adding the variance components estimated from random individuals into the genomic prediction of individuals with extreme phenotypes is expected to further improve prediction accuracy with selective genotyping.

The benefit of crossbreeding is the use of breed complementarity and heterosis [43, 58]. Compared with the PB reference population, the commercial CB population is not influenced by ${r}_{pc}$, and heterosis is only present in CB performance. Non-additive genetic effects, such as dominance and epistasis effects are the main causes of heterosis [59]. The benefits from CCPS when dominance is present are increased when dominance effects are included in the prediction model [20]. In our study, including dominance effects in the prediction model would reduce the accuracy of GEBV, as only additive effects were simulated. Previous results suggested that it would be better to include dominance and epistatic effects in the model, even if the action of nearly all the genes was additive, and dominance may be small or absent [19]. We believe that the performance of commercial crossbreds will be further enhanced by including non-additive effects in the prediction model due to the presence of heterosis in practice, especially for the low-heritability traits resulting from non-additive effects.

### Design of an optimal reference population

We explored the design of an optimal reference population for different breeds based on the prediction accuracy for each breed in the candidate group. When the GBLUP model is used and the environmental variance of the trait is small, the genetic relatedness between populations can provide a basis for the design of the reference population [45, 48]. In our study, the prediction accuracy within each breed and the genetic relatedness between the reference group and the corresponding breed were correlated when predicting a high-heritability trait with GBLUP. For a three-way crossbreeding system, the selection of terminal males is crucial since it accounts for half of the genetics of CB progeny [13]. Our results validated the high genetic relatedness between DLY and Duroc and demonstrated the superiority of CB performance in predicting Duroc candidates compared to PB performance, especially the CB performance from individuals from the top and bottom of the phenotype distribution. This indicated that crossbreds with extreme phenotypes in the commercial population constitute the optimum reference population for the selection of terminal paternal purebreds.

Concerning purebreds serving as the first sire, i.e., Landrace in this crossbreeding system, PB and CB populations showed similar genetic relatedness with Landrace candidates, which is consistent with the predicted results for Landrace candidates when using GBLUP (${h}^{2}$ = 0.5), for which the predictive performances of PB and CB were comparable. However, we found that BSLMM can significantly improve the prediction accuracy when using the CB_extreme reference population, with the maximum improvement in accuracy reaching 0.112 (reference population size = 6500), while the maximum improvement for PB was only 0.038 (reference population size = 2000). In addition, the CB_extreme reference population consistently outperformed PB for medium- and low-heritability traits, using either GBLUP or BSLMM, but BSLMM achieved a higher prediction accuracy. A reference population consisting of crossbreds in the commercial population with extreme phenotypes has a definite advantage for the prediction of the first sire, and the best selection response can be obtained when using BSLMM for prediction.

There is a great demand for purebreds serving as the first dam in three-way crossbreeding systems, such as Yorkshire in the present study. For high-heritability traits, the PB reference population was preferred to the CB reference population because a large number of Yorkshire individuals in the PB population resulted in a more similar LD pattern and, thus, higher genetic relatedness with Yorkshire candidates than the CB population [46]. The effect of genetic relatedness on prediction accuracy decreased as the environmental variance increased. Therefore, the prediction accuracy of PB for predicting Yorkshire candidates decreased significantly as the heritability decreased. However, since CB are not affected by ${r}_{pc}$, and the CB_extreme can provide an accurate estimate of marker effects [13], the CB_extreme population was superior to PB for the prediction of Yorkshire GEBV for medium- and low-heritability traits. For medium- or low-heritability traits, CB_extreme combined with BSLMM will be a good option for predicting the first dam.

Analysis of the size of the reference population indicated that an extreme sampling size of 4000 for CB performance in combination with the BSLMM model was adequate to carry out accurate predictions for medium-heritability traits. In contrast, a larger sampling size of 5000 was required for low-heritability traits.

### Reasons for the advantage of using extreme phenotypes for prediction

Selective genotyping was first applied in quantitative genetics by [60], and its efficiency in analyzing marker-QTL linkage was demonstrated subsequently [44]. A selective genotyping strategy with individuals that deviate from the population mean showed great potential for detecting genetic variation associated with quantitative traits [61–63]. The present study also confirmed the advantage of extreme selective genotyping (CB_extreme) for QTL identification, as the number of significant SNPs in high LD with QTL was larger and the PVE of significant peak SNPs was higher than for GWAS using the two random groups (PB and CB_random) given the same number of genotyped individuals. The reason is that more beneficial genetic information for QTL detection is available for individuals with extreme phenotypes, i.e. the density of trait-related genetic variation was greater with extreme selective genotyping and thus the frequency of mutant alleles at QTL was higher than with random genotyping [29]. Simulated QTL effects in this study were assumed to follow a Gamma distribution, resulting in relatively large effects for a small fraction of QTL [2, 33]. The GWAS that was carried out in an extreme subset of the population yielded more accurate localizations of large-effect QTL and a higher detection probability of small-effect QTL. Therefore, extreme selective genotyping can provide more accurate estimates of marker effects due to the LD between markers and QTL, which is the core of the BSLMM approach [1, 64].

The predicted results are based on simulated traits controlled by 2000 QTL. We also explored the effect of PB and CB reference populations for predicting traits controlled by much fewer QTL. Similarly, phenotypes for three traits controlled by 100 QTL corresponding to different heritabilities (${h}^{2}$ = 0.1, 0.3, and 0.5) were simulated. We compared the prediction accuracy of different reference scenarios for traits with a simpler genetic structure under the two models (see Additional file 9: Fig. S5). The results showed that even if the number of QTL for quantitative traits was small, higher prediction accuracy was obtained with the BSLMM model than with the GBLUP model, and the improvement in accuracy was most significant for the CB_extreme reference population. The combination of extreme selection genotyping and the BSLMM model is advantageous for genomic prediction regardless of the number of QTL associated with the trait. The ability of a population to detect QTL for a single trait indicates its effectiveness for predictions based on marker effects. We demonstrated that individuals with extreme phenotypes in the commercial population combined with BSLMM yielded the maximum selection response. The number of SNPs used for prediction in this experiment was only 37,755 and we believe that the combination of extreme CB performance and BSLMM will benefit more from increasing marker density and that the prediction accuracy of using commercial crossbreds with extreme phenotypes as a reference population will be further improved.

### Practicality in breeding farms

Many animal breeding companies will be reluctant to stop phenotyping at the PB level, and an important reason for this is the uncertainty in the CB information flow. The CB phenotypes must come from commercial farms and there is no guarantee that such farms will continue to phenotype their animals, especially for small breeding companies. They may go out of business or break down with an infectious disease and be unable to record all the required phenotypic information on the commercial population. Interruption of CB information flow from commercial farms would affect the company's breeding decisions. Our results demonstrate that genotyping crossbreds with extreme phenotypes and using them for GS prediction would be a good option. This idea could be picked up by breeding companies, but given the uncertainty in CB information, they will still depend on some PB information and likely use a combination of PB and CB. However, unlike the CCPS strategy in the past, they should replace data from random CB animals with data from CB animals with extreme phenotypes for the CB information flow they have.

Therefore, we tested the prediction accuracy of the CCPS system with the combination of PB and CB_extreme based on the simulated crossbreeding system (see Additional file 10: Fig. S6). Here, the proportion of CB to be included in the reference group is 50%. The results showed that the prediction accuracy of this CCPS strategy was consistently lower than that of PB and CB_extreme for the three traits with different heritabilities when using the GBLUP model. However, we found that the prediction accuracy of CCPS for high-heritability traits could be significantly improved to a level in between that reached by PB and CB_extreme when using the BSLMM model. For medium-heritability traits, for which the trends in prediction accuracy of PB and CB_extreme almost overlapped, the prediction accuracy of CCPS was slightly lower than that of the other two. For low-heritability traits, the results of prediction accuracy for the three reference populations were CB_extreme > PB > CCPS.

We have proved that CB_extreme has a definite advantage for the prediction of breeding values of PB candidates for medium- and low-heritability traits (Fig. 2 and Table. 2). However, this CCPS strategy can also accurately predict high-heritability traits, because the PB information that is included in this strategy could provide high genetic relatedness with PB candidates. In addition, the requirements for genetic relatedness between the reference and candidate populations are greater as heritability increases [45, 46]. Thus, if the CB information flow is incomplete in practical breeding, partial PB information can be included and combined with the available extreme CB information. This CCPS system (PB + CB_extreme) would be a good choice for the prediction of PB for CB performance for high-heritability traits. However, as heritability decreases, using full extreme CB information for genomic prediction is more effective.

## Conclusions

In this study, we simulated a three-way crossbreeding system of 100,000 DLY based on actual PB ancestors and explored the efficiency of using the commercial CB population for genomic prediction of breeding values of PB for CB performance. The predictive performance of using CB and PB data was compared across different population sizes and prediction models, providing a guide to the design of reference populations for traits of different heritabilities and for PB selection of different breeds. The results indicated that the performance of extreme CB has the potential to replace PB performance and in combination with the BSLMM model can maximize the response to selection. Genetic relatedness provided preliminary insights into the accuracy obtained for different reference populations under the GBLUP model. Moreover, the GWAS results demonstrated the high potential of commercial crossbreds with extreme phenotypes for QTL detection, which was correlated with its good predictive performance under the BSLMM model. For high-heritability traits, the results of extreme CB performance and PB performance were comparable when the influence of ${r}_{pc}$ on the accuracy obtained with a PB reference population was considered. The use of extreme CB data even exceeded that of using PB data in terms of response to selection at a large population size. For traits of medium-heritability, an extreme sample size of 4000 was sufficient to carry out accurate predictions of breeding values of PB candidates, but the sample size had to be increased to 5000 for low-heritability traits in order to achieve adequate accuracy. In a three-way crossbreeding system, extreme CB performance was superior to PB performance for the selection of purebreds that served as the first sire and terminal sire, while the optimal design of the reference group for the first dam depended on the heritability of the target trait and the percentage of purebreds from the corresponding breed that the PB reference data comprised. These findings present new guidance for the implementation of genomic selection in pig crossbreeding systems.

## Supplementary Information

**Additional file 1: Figure S1.** Predicted accuracy of estimated breeding values within each pure breed of the candidate population for different reference population types (PB: purebreds in GP2; CB_extreme: two_tailed crossbreds in DLY; CB_random: random crossbreds in DLY) and sizes (500, 1000, 2000, 3000, 4000, 5000, 6000, 6500). The results are shown for a low-heritability trait (${h}^{2}$ = 0.1). The accuracies of prediction for Duroc (a and b), Landrace (c and d), and Yorkshire (e and f) within the candidate population are shown separately. The results of the GBLUP method (a, c, and e) and the BSLMM method (b, d, and f) are presented. Predicted accuracy was averaged across 50 replications for scenarios involving randomization.

**Additional file 2: Figure S2.** Pairwise IBS calculations between the reference population and candidate population. The violin distribution of mean IBS coverage of all individuals in the reference population against each individual in the candidate population is shown. The x-axis denotes the breeds within the candidate population, and the y-axis is the mean IBS corresponding to each individual in the candidate population. The results for all reference population sizes (from 500 to 6500) and three reference population types (PB, CB_extreme, and CB_random) are shown. The IBS coverage for each individual was averaged across 50 replications for scenarios involving randomization.

**Additional file 3: Figure S3.** Pairwise IBD calculations between the reference population and GGP1 ancestral population (a and b), and between the reference population and candidate population (c and d). The scattered distribution of mean IBD coverage of all individuals in the reference population against each individual in the target population (GGP1/Candidate) is shown. The x-axis refers to each individual in the target population divided by breed, and the y-axis is the mean IBD corresponding to the targeted breed. The numbers of individuals in the GGP1 and candidate population were 690 and 2600, respectively, in which the breed ratios were 1:2:10 (Duroc: Landrace: Yorkshire) for both populations. The distributions of the three reference population types (PB, CB_extreme, and CB_random) and two population sizes (500 for a and c, 6500 for b, and d) are shown. The IBD coverage for each individual was averaged across 50 replications for scenarios involving randomization.

**Additional file 4: Figure S4.** Number of SNPs significantly associated with the simulated traits in different reference population scenarios detected by GWAS (a and c) and number of significant SNPs with an ${\mathrm{r}}^{2}$ higher than 0.6 (b and d). Results are presented for traits with ${h}^{2}$ = 0.3 (a and b) and 0.1 (c and d), including the investigations of three reference population types (PB, CB_extreme, and CB_random) and eight reference population sizes (500, 1000, 2000, 3000, 4000, 5000, 6000, 6500). The scenarios involving randomization were averaged, and error bars denotes the SD between 50 replicates.

**Additional file 5: Table S1.** Average TBV of the top 10% candidates ranked by GEBV for each pure breed in the candidate group predicted by different reference populations (PB: purebreds in GP2; CB_extreme: two_tailed crossbreds in DLY; CB_random: random crossbreds in DLY) with different population sizes (500, 1000, 2000, 3000, 4000, 5000, 6000, 6500) in the GBLUP model. TBV were averaged across 50 replications for scenarios involving randomization.

**Additional file 6: Table S2.** Average TBV of the top 10% candidates ranked by GEBV for each pure breed in the candidate group predicted by different reference populations (PB: purebreds in GP2; CB_extreme: two_tailed crossbreds in DLY; CB_random: random crossbreds in DLY) with different population sizes (500, 1000, 2000, 3000, 4000, 5000, 6000, 6500) in the BSLMM model. TBV were averaged across 50 replications for scenarios involving randomization.

**Additional file 7: Table S3.** Sum of the phenotypic variances explained (PVE) by the peak SNP within each QTL region in the GWAS results of different reference populations (PB: purebreds in GP2; CB_extreme: two_tailed crossbreds in DLY; CB_random: random crossbreds in DLY) with different population sizes (500, 1000, 2000, 3000, 4000, 5000, 6000, 6500) for a low-heritability trait (${h}^{2}$ = 0.1) (%). PVE were averaged across 50 replications for scenarios involving randomization.

**Additional file 8: Table S4.** Variance components estimated from the GBLUP model for different reference population scenarios. Vg: estimated genetic variance, Ve: estimated residual variance; variance was averaged across 50 replications for scenarios involving randomization.

**Additional file 9: Figure S5.** Predicted accuracy of estimated breeding values of the whole candidate population for different reference population types (PB: purebreds in GP2; CB_extreme: two_tailed crossbreds in DLY; CB_random: random crossbreds in DLY) and sizes (500, 1000, 2000, 3000, 4000, 5000, 6000, 6500). The predicted results are presented for the simulated traits controlled by 100 QTL. (a) GBLUP method, ${h}^{2}$ = 0.5, (b) BSLMM method, ${h}^{2}$ = 0.5, (c) GBLUP method, ${h}^{2}$ = 0.3, (d) BSLMM method, ${h}^{2}$ = 0.3, (e) GBLUP method, ${h}^{2}$ = 0.1, (f) BSLMM method, ${h}^{2}$ = 0.1. Predicted accuracy was averaged across 50 replications for scenarios involving randomization.

**Additional file 10: Figure S6.** Predicted accuracy of estimated breeding values of the whole candidate population for different reference population types (PB: purebreds in GP2; CB_extreme: two_tailed crossbreds in DLY; CCPS: PB + CB_extreme, 50% each) and sizes (500, 1000, 2000, 3000, 4000, 5000, 6000, 6500). (a) GBLUP method, ${h}^{2}$ = 0.5, (b) BSLMM method, ${h}^{2}$ = 0.5, (c) GBLUP method, ${h}^{2}$ = 0.3, (d) BSLMM method, ${h}^{2}$ = 0.3, (e) GBLUP method, $\small {h}^{2}$ = 0.1, (f) BSLMM method, ${h}^{2}$ = 0.1. Predicted accuracy was averaged across 50 replications for scenarios involving randomization.

## Data Availability

The source data and scripts for simulation are available in the repository: https://github.com/April-641/Genomic-Prediction.
