# Supplementary figures and images for: Genomic prediction in pigs using data from a commercial crossbred population: insights from the Duroc x (Landrace x Yorkshire) three-way crossbreeding system
Source: Genet Sel Evol. 2023 Mar 28;55:21. doi: 10.1186/s12711-023-00794-2 (PMC10053053; doi:10.1186/s12711-023-00794-2)

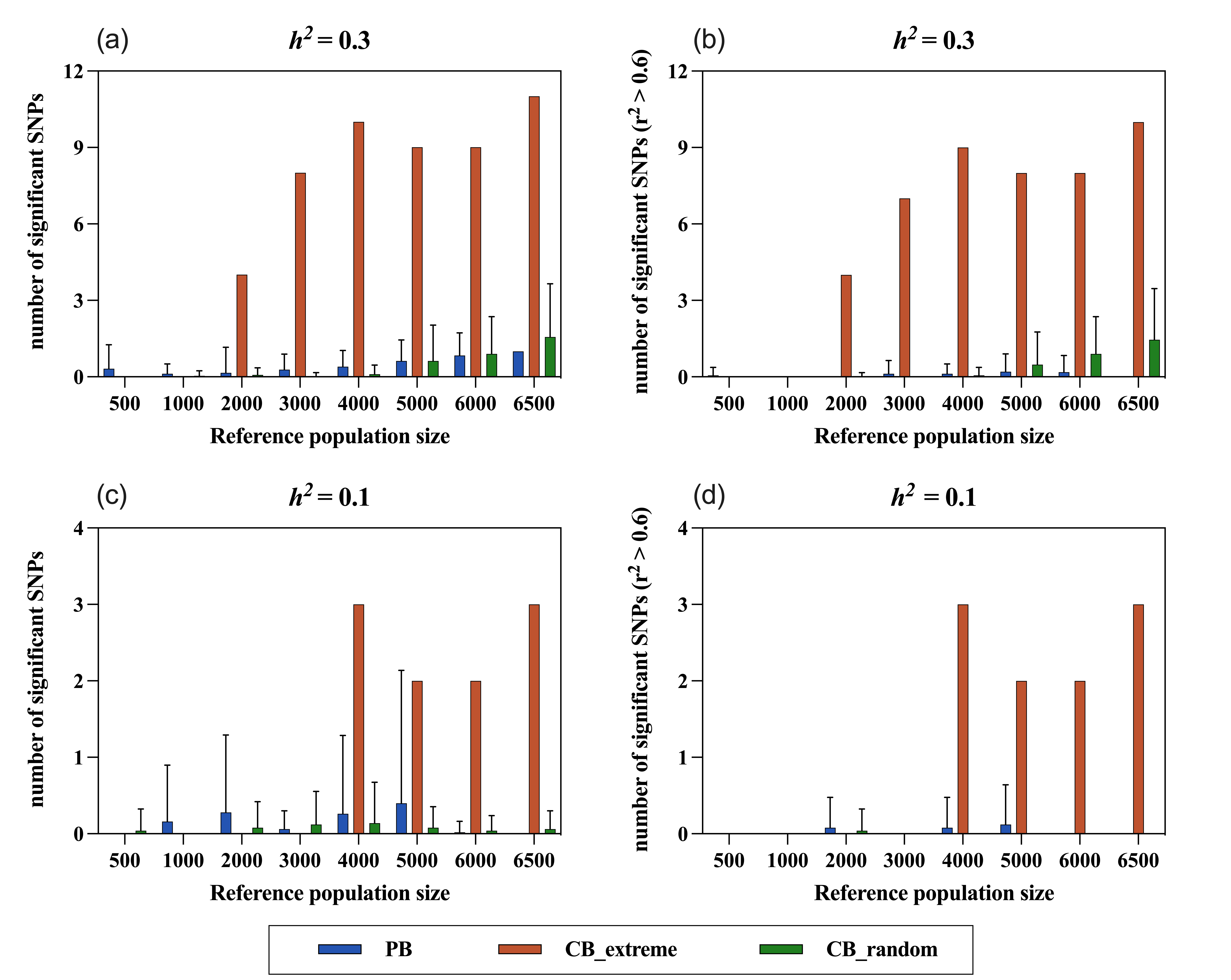

Supplement: Supplementary file 4 — Additional file 4: Figure S4. Number of SNPs significantly associated with the simulated traits in different reference population scenarios detected by GWAS (a and c) and number of significant SNPs with an ${\mathrm{r}}^{2}$r2 higher than 0.6 (b and d). Results are presented for traits with ${h}^{2}$h2 = 0.3 (a and b) and 0.1 (c and d), including the investigations of three reference population types (PB, CB_extreme, and CB_random) and eight reference population sizes (500, 1000, 2000, 3000, 4000, 5000, 6000, 6500). The scenarios involving randomization were averaged, and error bars denotes the SD between 50 replicates. [file 12711_2023_794_MOESM4_ESM.tiff]
