# Supplementary material for: Genomic prediction in pigs using data from a commercial crossbred population: insights from the Duroc x (Landrace x Yorkshire) three-way crossbreeding system
Source: Genet Sel Evol. 2023 Mar 28;55:21. doi: 10.1186/s12711-023-00794-2 (PMC10053053; doi:10.1186/s12711-023-00794-2)
Supplement: Supplementary file 5 — Additional file 5: Table S1. Average TBV of the top 10% candidates ranked by GEBV for each pure breed in the candidate group predicted by different reference populations (PB: purebreds in GP2; CB_extreme: two_tailed crossbreds in DLY; CB_random: random crossbreds in DLY) with different population sizes (500, 1000, 2000, 3000, 4000, 5000, 6000, 6500) in the GBLUP model. TBV were averaged across 50 replications for scenarios involving randomization. [file 12711_2023_794_MOESM5_ESM.docx]

**Table S1** **Average TBV of the top 10% candidates ranked by GEBV for each pure breed in the candidate group predicted by different reference populations** **(PB: purebreds in GP2; CB_extreme: two_tailed crossbreds in DLY; CB_random: random crossbreds in DLY) with different population sizes (500, 1000, 2000, 3000, 4000, 5000, 6000, 6500) in the GBLUP model**

| $\boldsymbol{h}^{\boldsymbol{2}}$ | **class** | **Candidate_Duroc** | | | | **Candidate_Landrace** | | | | **Candidate_Yorkshire** | | |
| --- | --- | --- | --- | --- | --- | --- | --- | --- | --- | --- | --- | --- |
|  |  | **PB** | **CB_extreme** | **CB_random** | **PB** | | **CB_extreme** | **CB_random** | **PB** | | **CB_****extreme** | **CB_****random** |
| 0.5 | 6500 | 3.023 | 3.357 | 3.198 | 3.264 | | 3.258 | 3.147 | 2.773 | | 2.602 | 2.483 |
|  | 6000 | 2.901 | 3.319 | 3.178 | 3.252 | | 3.154 | 3.161 | 2.764 | | 2.589 | 2.489 |
|  | 5000 | 2.959 | 3.299 | 3.125 | 3.222 | | 3.275 | 3.113 | 2.728 | | 2.642 | 2.439 |
|  | 4000 | 2.943 | 3.209 | 3.138 | 3.171 | | 3.314 | 3.089 | 2.688 | | 2.546 | 2.379 |
|  | 3000 | 2.925 | 3.283 | 3.086 | 3.117 | | 3.188 | 3.032 | 2.635 | | 2.482 | 2.333 |
|  | 2000 | 2.856 | 3.035 | 3.064 | 3.045 | | 3.060 | 2.977 | 2.558 | | 2.425 | 2.263 |
|  | 1000 | 2.704 | 3.305 | 3.018 | 2.923 | | 2.801 | 2.860 | 2.375 | | 2.312 | 2.145 |
|  | 500 | 2.534 | 3.162 | 2.934 | 2.804 | | 3.278 | 2.768 | 2.241 | | 2.240 | 2.057 |
| 0.3 | 6500 | 3.161 | 3.267 | 3.020 | 3.051 | | 3.048 | 2.958 | 2.547 | | 2.423 | 2.217 |
|  | 6000 | 3.110 | 3.282 | 3.014 | 2.983 | | 3.072 | 2.964 | 2.520 | | 2.453 | 2.214 |
|  | 5000 | 2.992 | 3.091 | 2.978 | 2.909 | | 3.096 | 2.961 | 2.484 | | 2.372 | 2.184 |
|  | 4000 | 2.912 | 3.181 | 2.980 | 2.817 | | 3.092 | 2.882 | 2.424 | | 2.326 | 2.134 |
|  | 3000 | 2.848 | 3.105 | 2.943 | 2.768 | | 3.075 | 2.793 | 2.346 | | 2.238 | 2.115 |
|  | 2000 | 2.654 | 3.152 | 2.894 | 2.744 | | 3.056 | 2.771 | 2.274 | | 2.208 | 2.069 |
|  | 1000 | 2.455 | 3.255 | 2.850 | 2.593 | | 2.738 | 2.678 | 2.121 | | 2.234 | 2.011 |
|  | 500 | 2.328 | 2.933 | 2.620 | 2.481 | | 2.625 | 2.604 | 2.037 | | 2.135 | 1.874 |
| 0.1 | 6500 | 2.116 | 3.018 | 2.745 | 2.680 | | 3.093 | 2.633 | 1.795 | | 2.123 | 1.869 |
|  | 6000 | 2.067 | 3.018 | 2.665 | 2.719 | | 3.084 | 2.653 | 1.765 | | 2.037 | 1.866 |
|  | 5000 | 2.090 | 2.992 | 2.729 | 2.709 | | 2.974 | 2.614 | 1.763 | | 2.073 | 1.865 |
|  | 4000 | 2.028 | 2.923 | 2.685 | 2.675 | | 3.004 | 2.535 | 1.796 | | 2.004 | 1.798 |
|  | 3000 | 2.053 | 2.811 | 2.580 | 2.673 | | 2.914 | 2.590 | 1.790 | | 1.965 | 1.783 |
|  | 2000 | 2.202 | 2.996 | 2.465 | 2.702 | | 2.734 | 2.517 | 1.773 | | 1.915 | 1.711 |
|  | 1000 | 2.151 | 2.876 | 2.369 | 2.520 | | 2.632 | 2.465 | 1.694 | | 1.931 | 1.587 |
|  | 500 | 2.147 | 2.860 | 2.302 | 2.485 | | 2.459 | 2.404 | 1.661 | | 1.884 | 1.586 |

The TBV was averaged across 50 replications for scenarios involving randomization
