# Supplementary material for: Genomic prediction in pigs using data from a commercial crossbred population: insights from the Duroc x (Landrace x Yorkshire) three-way crossbreeding system
Source: Genet Sel Evol. 2023 Mar 28;55:21. doi: 10.1186/s12711-023-00794-2 (PMC10053053; doi:10.1186/s12711-023-00794-2)
Supplement: Supplementary file 6 — Additional file 6: Table S2. Average TBV of the top 10% candidates ranked by GEBV for each pure breed in the candidate group predicted by different reference populations (PB: purebreds in GP2; CB_extreme: two_tailed crossbreds in DLY; CB_random: random crossbreds in DLY) with different population sizes (500, 1000, 2000, 3000, 4000, 5000, 6000, 6500) in the BSLMM model. TBV were averaged across 50 replications for scenarios involving randomization. [file 12711_2023_794_MOESM6_ESM.docx]

**Table S2** **Average TBV of the top 10% candidates ranked by GEBV for each pure breed in the candidate group predicted by different reference populations (PB: purebreds in GP2; CB_extreme: two_tailed crossbreds in DLY; CB_random: random crossbreds in DLY) with different population sizes (500, 1000, 2000, 3000, 4000, 5000, 6000, 6500) in the BSLMM model**

| $\boldsymbol{h}^{\boldsymbol{2}}$ | **class** | **Candidate_Duroc** | | | **Candidate_Landrace** | | | **Candidate_Yorkshire** | | |
| --- | --- | --- | --- | --- | --- | --- | --- | --- | --- | --- |
|  |  | **PB** | **CB_extreme** | **CB_random** | **PB** | **CB_extreme** | **CB_random** | **PB** | **CB_extreme** | **CB_random** |
| 0.5 | 6500 | 3.337 | 3.423 | 3.297 | 3.304 | 3.437 | 3.302 | 2.835 | 2.741 | 2.615 |
|  | 6000 | 3.309 | 3.408 | 3.294 | 3.294 | 3.420 | 3.296 | 2.822 | 2.733 | 2.611 |
|  | 5000 | 3.236 | 3.390 | 3.253 | 3.300 | 3.339 | 3.246 | 2.796 | 2.759 | 2.564 |
|  | 4000 | 3.190 | 3.365 | 3.253 | 3.253 | 3.311 | 3.188 | 2.768 | 2.706 | 2.508 |
|  | 3000 | 3.083 | 3.308 | 3.207 | 3.194 | 3.389 | 3.158 | 2.711 | 2.671 | 2.444 |
|  | 2000 | 2.934 | 3.235 | 3.129 | 3.109 | 3.152 | 3.071 | 2.618 | 2.516 | 2.333 |
|  | 1000 | 2.735 | 3.210 | 3.042 | 2.948 | 3.065 | 2.896 | 2.398 | 2.406 | 2.165 |
|  | 500 | 2.519 | 3.295 | 2.914 | 2.795 | 3.130 | 2.746 | 2.248 | 2.257 | 2.040 |
| 0.3 | 6500 | 3.043 | 3.399 | 3.109 | 2.846 | 3.285 | 3.041 | 2.603 | 2.583 | 2.262 |
|  | 6000 | 3.087 | 3.167 | 3.048 | 3.011 | 3.250 | 3.031 | 2.563 | 2.571 | 2.252 |
|  | 5000 | 3.067 | 3.335 | 3.051 | 2.953 | 3.254 | 2.987 | 2.520 | 2.566 | 2.225 |
|  | 4000 | 2.954 | 3.249 | 2.999 | 2.863 | 3.183 | 2.904 | 2.449 | 2.475 | 2.144 |
|  | 3000 | 2.870 | 3.205 | 2.955 | 2.781 | 3.226 | 2.812 | 2.359 | 2.392 | 2.123 |
|  | 2000 | 2.693 | 3.192 | 2.903 | 2.755 | 3.150 | 2.761 | 2.288 | 2.271 | 2.062 |
|  | 1000 | 2.458 | 3.295 | 2.847 | 2.581 | 2.807 | 2.658 | 2.118 | 2.249 | 2.005 |
|  | 500 | 2.324 | 2.933 | 2.632 | 2.504 | 2.623 | 2.637 | 2.040 | 2.131 | 1.829 |
| 0.1 | 6500 | 1.958 | 3.083 | 2.769 | 2.787 | 3.073 | 2.579 | 1.886 | 2.266 | 1.841 |
|  | 6000 | 2.047 | 3.205 | 2.647 | 2.741 | 3.166 | 2.603 | 1.868 | 2.237 | 1.826 |
|  | 5000 | 2.094 | 3.176 | 2.690 | 2.717 | 3.064 | 2.600 | 1.858 | 2.218 | 1.814 |
|  | 4000 | 2.106 | 3.126 | 2.672 | 2.678 | 2.935 | 2.494 | 1.859 | 2.056 | 1.732 |
|  | 3000 | 2.110 | 2.978 | 2.557 | 2.664 | 2.952 | 2.556 | 1.843 | 2.054 | 1.722 |
|  | 2000 | 2.264 | 2.996 | 2.458 | 2.671 | 2.738 | 2.451 | 1.800 | 1.939 | 1.670 |
|  | 1000 | 2.149 | 2.892 | 2.395 | 2.524 | 2.644 | 2.429 | 1.722 | 1.889 | 1.535 |
|  | 500 | 2.150 | 2.714 | 2.318 | 2.477 | 2.539 | 2.370 | 1.665 | 1.844 | 1.544 |

The TBV was averaged across 50 replications for scenarios involving randomization
