# Supplementary material for: Genomic prediction in pigs using data from a commercial crossbred population: insights from the Duroc x (Landrace x Yorkshire) three-way crossbreeding system
Source: Genet Sel Evol. 2023 Mar 28;55:21. doi: 10.1186/s12711-023-00794-2 (PMC10053053; doi:10.1186/s12711-023-00794-2)
Supplement: Supplementary file 7 — Additional file 7: Table S3. Sum of the phenotypic variances explained (PVE) by the peak SNP within each QTL region in the GWAS results of different reference populations (PB: purebreds in GP2; CB_extreme: two_tailed crossbreds in DLY; CB_random: random crossbreds in DLY) with different population sizes (500, 1000, 2000, 3000, 4000, 5000, 6000, 6500) for a low-heritability trait (${h}^{2}$h2 = 0.1) (%). PVE were averaged across 50 replications for scenarios involving randomization. [file 12711_2023_794_MOESM7_ESM.docx]

**Table S3 Sum of the phenotypic variances explained (PVE) by the peak SNP within each QTL region in the GWAS results of different reference populations (PB: purebreds in GP2; CB_extreme: two_tailed crossbreds in DLY; CB_random: random** **crossbreds in DLY) with different population sizes (500, 1000, 2000, 3000, 4000, 5000, 6000, 6500) for a low-heritability trait (**$\boldsymbol{h}^{\boldsymbol{2}}$ **= 0.1) (%)**

| $\boldsymbol{h}^{\boldsymbol{2}}$ | **class** | **reference population size** | | | | | | | |
| --- | --- | --- | --- | --- | --- | --- | --- | --- | --- |
|  |  | **500** | **1000** | **2000** | **3000** | **4000** | **5000** | **6000** | **6500** |
| 0.1 | PB | 0 | 0.046 | 0.075 | 0 | 0.027 | 0.029 | 0 | 0 |
|  | CB_extreme | 0 | 0 | 0 | 0 | 0.810 | 0.541 | 0.420 | 0.390 |
|  | CB_random | 0 | 0 | 0.027 | 0.016 | 0 | 0 | 0 | 0 |

The PVE was averaged across 50 replications for scenarios involving randomization
