# Supplementary material for: Genomic prediction in pigs using data from a commercial crossbred population: insights from the Duroc x (Landrace x Yorkshire) three-way crossbreeding system
Source: Genet Sel Evol. 2023 Mar 28;55:21. doi: 10.1186/s12711-023-00794-2 (PMC10053053; doi:10.1186/s12711-023-00794-2)
Supplement: Supplementary file 8 — Additional file 8: Table S4. Variance components estimated from the GBLUP model for different reference population scenarios. Vg: estimated genetic variance, Ve: estimated residual variance; variance was averaged across 50 replications for scenarios involving randomization. [file 12711_2023_794_MOESM8_ESM.docx]

**Table S4 Variance components estimated from GBLUP model for different reference population scenarios**

| **reference population size** | **class** | $\boldsymbol{h}^{\boldsymbol{2}}$ **= 0.5** | | $\boldsymbol{h}^{\boldsymbol{2}}$ **= 0.3** | | $\boldsymbol{h}^{\boldsymbol{2}}$ **= 0.1** | |
| --- | --- | --- | --- | --- | --- | --- | --- |
|  |  | $\boldsymbol{V}_{\boldsymbol{g}}$ | $\boldsymbol{V}_{\boldsymbol{e}}$ | $\boldsymbol{V}_{\boldsymbol{g}}$ | $\boldsymbol{V}_{\boldsymbol{e}}$ | $\boldsymbol{V}_{\boldsymbol{g}}$ | $\boldsymbol{V}_{\boldsymbol{e}}$ |
| 6500 | PB | 1.005 | 1.025 | 0.876 | 5.352 | 0.603 | 80.253 |
|  | CB_extreme | 5.006 | 1.353 | 15.183 | 17.075 | 30.606 | 387.951 |
|  | CB_random | 0.973 | 1.047 | 1.060 | 5.487 | 0.997 | 80.341 |
| 6000 | PB | 1.009 | 1.024 | 0.874 | 5.349 | 0.548 | 80.352 |
|  | CB_extreme | 5.192 | 1.317 | 16.031 | 17.116 | 31.321 | 398.462 |
|  | CB_random | 0.991 | 1.042 | 1.044 | 5.488 | 1.193 | 79.928 |
| 5000 | PB | 1.007 | 1.022 | 0.879 | 5.352 | 0.506 | 80.326 |
|  | CB_extreme | 5.611 | 1.203 | 17.485 | 17.505 | 36.740 | 419.789 |
|  | CB_random | 0.989 | 1.043 | 1.051 | 5.498 | 1.471 | 80.203 |
| 4000 | PB | 1.013 | 1.019 | 0.878 | 5.350 | 0.606 | 79.988 |
|  | CB_extreme | 6.169 | 1.062 | 19.645 | 17.954 | 49.349 | 440.506 |
|  | CB_random | 1.018 | 1.031 | 1.046 | 5.485 | 1.204 | 80.130 |
| 3000 | PB | 1.008 | 1.029 | 0.860 | 5.387 | 0.427 | 79.784 |
|  | CB_extreme | 6.841 | 0.881 | 23.250 | 17.619 | 51.655 | 478.968 |
|  | CB_random | 1.021 | 1.026 | 1.046 | 5.542 | 1.149 | 79.553 |
| 2000 | PB | 1.002 | 1.030 | 0.878 | 5.355 | 0.575 | 79.336 |
|  | CB_extreme | 7.998 | 0.520 | 28.958 | 16.816 | 64.701 | 526.481 |
|  | CB_random | 1.043 | 1.021 | 1.130 | 5.422 | 1.584 | 79.127 |
| 1000 | PB | 0.982 | 1.022 | 0.847 | 5.317 | 0.910 | 78.731 |
|  | CB_extreme | 11.889 | 0.001 | 46.206 | 10.782 | 76.239 | 617.992 |
|  | CB_random | 1.103 | 0.984 | 1.164 | 5.377 | 1.964 | 78.157 |
| 500 | PB | 0.990 | 1.032 | 0.917 | 5.298 | 1.081 | 77.356 |
|  | CB_extreme | 14.281 | 0.000 | 59.825 | 7.189 | 135.450 | 667.856 |
|  | CB_random | 1.185 | 0.925 | 1.190 | 5.406 | 3.470 | 77.500 |

$\boldsymbol{V}_{\boldsymbol{g}}$: the estimated genetic variance, $\boldsymbol{V}_{\boldsymbol{e}}$: the estimated residual variance

The variance was averaged across 50 replications for scenarios involving randomization
